# Soluble CD93 lectin-like domain sequesters HMGB1 to ameliorate inflammatory diseases

**DOI:** 10.7150/thno.84935

**Published:** 2023-07-14

**Authors:** Shang-En Huang, Cheng-Hsiang Kuo, Si-Yu Shiao, Chia-Rui Shen, Fang-Tzu Lee, Bi-Ing Chang, Jong-Hau Hsu, Hua-Lin Wu, Jwu-Lai Yeh, Chao-Han Lai

**Affiliations:** 1 Graduate Institute of Medicine, College of Medicine, Kaohsiung Medical University, Kaohsiung, Taiwan.; 2 International Center for Wound Repair and Regeneration, National Cheng Kung University, Tainan, Taiwan.; 3 Department of Biochemistry and Molecular Biology, College of Medicine, National Cheng Kung University, Tainan, Taiwan.; 4 Cardiovascular Research Center, National Cheng Kung University, Tainan, Taiwan.; 5 Department of Medical Biotechnology and Laboratory Science, College of Medicine, Chang Gung University, Taoyuan, Taiwan.; 6 Department of Surgery, National Cheng Kung University Hospital, College of Medicine, National Cheng Kung University, Tainan, Taiwan.; 7 Department of Pediatrics, Kaohsiung Medical University Hospital, Kaohsiung, Taiwan.; 8 Department of Pediatrics, College of Medicine, Kaohsiung Medical University, Kaohsiung, Taiwan.; 9 Department of Pharmacology, College of Medicine, Kaohsiung Medical University, Kaohsiung, Taiwan.; 10 Department of Medical Research, Kaohsiung Medical University Hospital, Kaohsiung, Taiwan.; 11 Department of Marine Biotechnology and Resources, National Sun Yat-sen University, Kaohsiung, Taiwan.; 12 Department of Biostatistics, Vanderbilt University Medical Center, Nashville, Tennessee, USA.

**Keywords:** CD93, high-mobility group box 1 (HMGB1) protein, inflammation, abdominal aortic aneurysm, osteoporosis

## Abstract

**Rationale:** CD93, a C-type lectin-like transmembrane glycoprotein, can be shed in a soluble form (sCD93) upon inflammatory stimuli. sCD93 effectively enhances apoptotic cell clearance and has been proposed as an inflammatory disease biomarker. The function of sCD93 involved directly in inflammation remains to be determined. Herein, we attempted to examine the hypothesis that sCD93 might sequester proinflammatory high-mobility group box 1 protein (HMGB1), exerting anti-inflammatory properties.

**Methods:** Different forms of soluble recombinant human CD93 (rCD93) were prepared by a mammalian protein expression system. rCD93-HMGB1 interaction was assessed using co-immunoprecipitation and solid-phase binding assays. Effects of soluble rCD93 were evaluated in HMGB1-induced macrophage and vascular smooth muscle cells (VSMC) activation and receptor activator of nuclear factor-κB ligand (RANKL)-induced osteoclastogenesis, CaCl_2_-induced and angiotensin II-infused abdominal aortic aneurysm (AAA) formation and ovariectomized-induced osteoporosis in mice.

**Results:** Protein binding studies revealed that soluble rCD93, via the lectin-like domain (D1), can bind to HMGB1 and intercept HMGB1-receptor interaction. Soluble rCD93 containing D1 inhibited HMGB1-induced proinflammatory cytokine production and intracellular mitogen-activated protein kinase (MAPK)/nuclear factor (NF)-κB activation in macrophages and VSMCs, thereby attenuating CaCl_2_-induced and angiotensin II-infused AAA models. During osteoclastogenesis, RANKL stimulated HMGB1 secretion that promoted RANKL-induced osteoclastogenesis in return. Soluble rCD93 containing D1 impeded RANKL-induced osteoclastogenic marker gene expression and intracellular MAPK/NF-κB signaling, thereby mitigating ovariectomized-induced osteoporosis.

**Conclusion:** These findings demonstrate the therapeutic potential of soluble recombinant CD93 containing D1 in inflammatory diseases. Our study highlights a novel anti-inflammatory mechanism, i.e., sequestration of HMGB1, through which sCD93 prevents HMGB1-receptor interaction on effector cells and alleviates inflammation.

## Introduction

CD93, prominently expressed on myeloid cells, platelets, and endothelial cells, was first characterized as a receptor for the complement component C1q 1, 2. CD93 belongs to the C‐type lectin-like domain group 14 family of transmembrane glycoproteins 2. Structurally, the extracellular portion of CD93 consists of an N-terminal lectin-like domain (designated as D1), a tandem array of five epidermal growth factor-like repeats (D2), and a serine/threonine-rich mucin-like domain (D3) 1-3. CD93 is involved in several physiological and pathological processes, including fibrillogenesis (i.e., the formation of collagen fibrils), angiogenesis, phagocytosis, cell adhesion, and migration 2, 4.

Upon inflammatory stimuli, membrane-bound (cell-associated) CD93 is shed from the cell surface 5, 6, suggesting that CD93 participates in inflammation. This cleavage event releases a truncated soluble form of CD93 (sCD93) and most likely liberates the entire ectodomain (D123) 5, 6. sCD93 effectively enhances the engulfment of apoptotic cells by monocytes/macrophages (efferocytosis) 6-8, during which sCD93 serves as the bridging of apoptotic cells to phagocyte-expressed α_x_β_2_ 8. Defective efferocytosis may predispose to impaired inflammation resolution 8-10; thus, sCD93 exhibits anti-inflammatory activity in this context. Also, sCD93 has been proposed as a disease biomarker, as evidenced by the correlation between the circulating levels of sCD93 and disease status of inflammatory diseases such as asthma exacerbation and coronary atherosclerosis 11-15. However, the function of sCD93 involved directly in inflammation remains to be determined.

High-mobility group box 1 (HMGB1) protein, a damage-associated molecular pattern (DAMP) molecule, is a highly conserved, nuclear architectural chromatin-binding protein present in all cell types 16. In an inflammatory milieu, HMGB1 can be actively secreted from inflammatory cells triggered by proinflammatory cytokines such as tumor necrosis factor (TNF)-α or passively released from necrotic cells 17-19. Engagement with surface receptors (e.g., receptor for advanced glycation end-product [RAGE]) by extracellular HMGB1 may trigger proinflammatory pathways, resulting in activation of nuclear factor (NF)-κB, production of proinflammatory cytokines and elaboration of oxidative stress 16, 19-22. HMGB1-receptor interaction can amplify inflammatory signaling with positive feedback reactions and propagate inflammatory effects to neighboring cells 18, 20, 22, leading to tissue injury and organ damage.

In the studies presented here, we attempted to investigate the hypothesis that sCD93 might interact with HMGB1, a crucial proinflammatory mediator, thereby preventing it from engaging its receptors (e.g., RAGE) and hampering downstream inflammatory signaling. For this purpose, soluble recombinant human CD93 (rCD93) ectodomain proteins were prepared. We examined whether soluble rCD93 binds to HMGB1 and suppresses HMGB1-induced inflammatory events. In addition, *in vivo* studies were performed to evaluate whether soluble rCD93 mitigates HMGB1-driven inflammatory diseases, including experimental CaCl_2_-induced and angiotensin II (AngII)-infused abdominal aortic aneurysm (AAA) and ovariectomized (OVX)-induced osteoporosis in mice. These findings may help gain insight into the functional significance of sCD93 in inflammatory disorders.

## Results

### Soluble rCD93 binds to HMGB1 via D1

**Figure 1:**
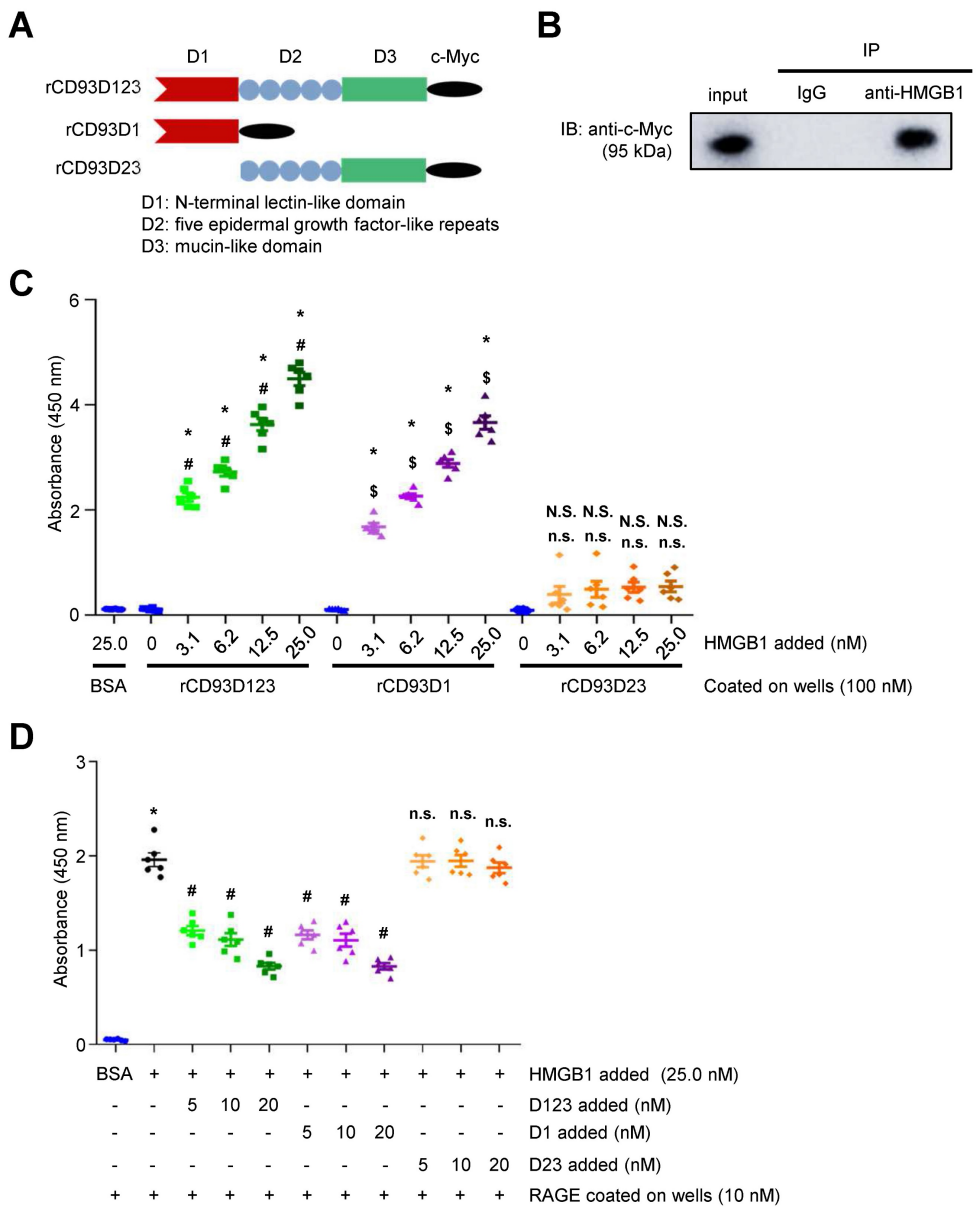
** Soluble rCD93, via D1, binds to HMGB1. (A)** Three soluble forms of purified recombinant human CD93 ectodomain proteins tagged with c-Myc. **(B)** Incubation of recombinant human HMGB1 and soluble rCD93D123 was followed by co-immunoprecipitation with anti-HMGB1 antibody or rabbit IgG control antibody (negative control). Immunoblotting of the immunoprecipitates was performed using anti-c-Myc antibody. Representative results from three independent experiments are shown. **(C)** The protein binding between HMGB1 and BSA (negative control) or soluble rCD93 ectodomain proteins was examined using ELISA solid-phase binding assay (n = 6). *P < 0.0001 vs. BSA-HMGB1 binding. For rCD93D123, ^#^*P* < 0.0001 vs. rCD93D123-HMGB1 (0 nM) binding. For rCD93D1, ^$^*P* < 0.0001 vs. rCD93D1-HMGB1 (0 nM) binding. For rCD93D23, N.S. *P* > 0.9999 rCD93D23-HMGB1 (3.1 nM) vs. BSA-HMGB1 binding; N.S. *P* = 0.5937 rCD93D23-HMGB1 (6.2 nM) vs. BSA-HMGB1 binding; N.S. *P* = 0.2472 rCD93D23-HMGB1 (12.5 nM) vs. BSA-HMGB1 binding; N.S. *P* = 0.1762 rCD93D23-HMGB1 (25.0 nM) vs. BSA-HMGB1 binding; n.s. *P* > 0.9999 rCD93D23-HMGB1 (3.1 nM) vs. rCD93D23-HMGB1 (0 nM) binding; n.s. *P* = 0.3946 rCD93D23-HMGB1 (6.2 nM) vs. rCD93D23-HMGB1 (0 nM) binding; n.s. *P* = 0.1601 rCD93D23-HMGB1 (12.5 nM) vs. rCD93D23-HMGB1 (0 nM) binding; n.s. *P* = 0.1131 rCD93D23-HMGB1 (25.0 nM) vs. rCD93D23-HMGB1 (0 nM) binding.** (D)** The competitive inhibition on HMGB1-RAGE binding by soluble rCD93 ectodomain proteins was examined using ELISA solid-phase binding assay (n =6). *P < 0.0001 vs. BSA-RAGE binding; ^#^*P* < 0.0001 vs. HMGB1-RAGE binding; n.s. *P* > 0.9999 vs. HMGB1-RAGE binding. D123, D1, and D23 indicate rCD93D123, rCD93D1, and rCD93D23, respectively. Data are represented as mean values ± SEM and comparative statistical analyses were done by one-way ANOVA followed by multiple comparisons.

CD93 shedding occurs upon inflammatory stimuli and most likely releases the entire ectodomain (D123) 5, 6. Three soluble forms of purified c-Myc-tagged rCD93 ectodomain proteins, rCD93D123, rCD93D1, and rCD93D23, were prepared by a mammalian protein expression system (**Figure 1A**). Herein, we explored whether soluble rCD93 binds to HMGB1, a necessary and sufficient inflammatory mediator in both sterile and infectious inflammation 16, 18, 19. Incubation of recombinant human HMGB1 and soluble rCD93D123 was followed by co-immunoprecipitation with anti-HMGB1 antibody and immunoblotting of the immunoprecipitates with anti-c-Myc antibody. An immunoreactive band of rCD93D123 was observed when anti-HMGB1 antibody was used for immunoprecipitation (**Figure 1B**) but not when a rabbit IgG control antibody was used, indicating that rCD93D123 may interact with HMGB1. Subsequently, the potential binding site for soluble rCD93 interaction with HMGB1 was explored. Assessment of rCD93D1, rCD93D23, and rCD93D123 using Coomassie blue staining and western blot analysis revealed that the molecular masses of rCD93D1, rCD93D23, and rCD93D123 were 20 kDa, 71 kDa, and 95 kDa, respectively (**Figure S1A**). Far-western blot analysis using HMGB1 as the probe, which was performed to evaluate rCD93-HMGB1 interaction, detected abundant expression of rCD93D1 (20 kDa) and rCD93D123 (95 kDa) but a scarce expression of rCD93D23 (71 kDa; **Figure S1B**), indicating D1 of soluble rCD93 as the possible binding site interacting with HMGB1.

The enzyme-linked immunosorbent assay (ELISA) solid-phase binding assay was performed to assess the direct binding of soluble rCD93 and HMGB1. In an assay with mole-equivalent rCD93D123, rCD93D1, and rCD93D23 immobilized on the plate, the binding of HMGB1 to rCD93D1 and rCD93D123 was increased by HMGB1 in a concentration-dependent manner, whereas the binding of HMGB1 and rCD93D23 was persistently low (**Figure 1C**). Consistently, in an assay with incremental concentrations of rCD93 proteins immobilized on the plate, the binding of HMGB1 to rCD93D1 and rCD93D123, but not rCD93D23, was increased in a concentration-dependent manner by rCD93 proteins (**Figure S1C**). RAGE is a signal transduction receptor expressed in great abundance on inflammatory cells (e.g., macrophages) 18, 23, 24 and mediates the proinflammatory effects of HMGB1 that lead to cellular activation 16, 19-22. Whether rCD93-HMGB1 binding affects HMGB1 interaction with RAGE and macrophages was explored. In a competitive binding assay with immobilized RAGE, adding rCD93D1 and rCD93D123 decreased HMGB1-RAGE binding in a dose-dependent manner, whereas adding rCD93D23 did not (**Figure 1D**). Soluble RAGE (sRAGE), containing the extracellular ligand-binding domain of RAGE 17, can serve as a decoy by binding HMGB1 and preventing its interaction with cell surface receptors 17, 24. In flow cytometry analysis, HMGB1 can bind to the RAW264.7 macrophage surface, and HMGB1 interaction with macrophages was inhibited by sRAGE (**Figure S1D**). Notably, HMGB1-macrophage interaction was inhibited by rCD93D1 and rCD93D123 but not rCD93D23. Flow cytometry analysis using fixed cells was performed to differentiate the possible effects between endocytic removal of rCD93-HMGB1 complexes and competition of rCD93 proteins with RAGE for HMGB1 binding. HMGB1 did not bind to the non-RAGE-expressing Chinese hamster ovary (CHO) cells (**Figure S2A**). In contrast, HMGB1 can bind to RAW264.7 macrophages, and HMGB1-macrophage interaction was inhibited by rCD93D1 and rCD93D123 but not rCD93D23 (**Figure S2B**), consistent with flow cytometry analysis using live cells. These observations suggested that rCD93D1 and rCD93D123 intercepted the interaction of HMGB1 with macrophages, on which RAGE is expressed.

Taken together, these protein binding studies suggested that soluble rCD93 can bind to HMGB1 and prevent its engagement with RAGE and macrophages, and their interaction may occur at the level of D1.

### Soluble rCD93 inhibits HMGB1-induced macrophage and vascular smooth muscle cell (VSMC) activation

**Figure 2:**
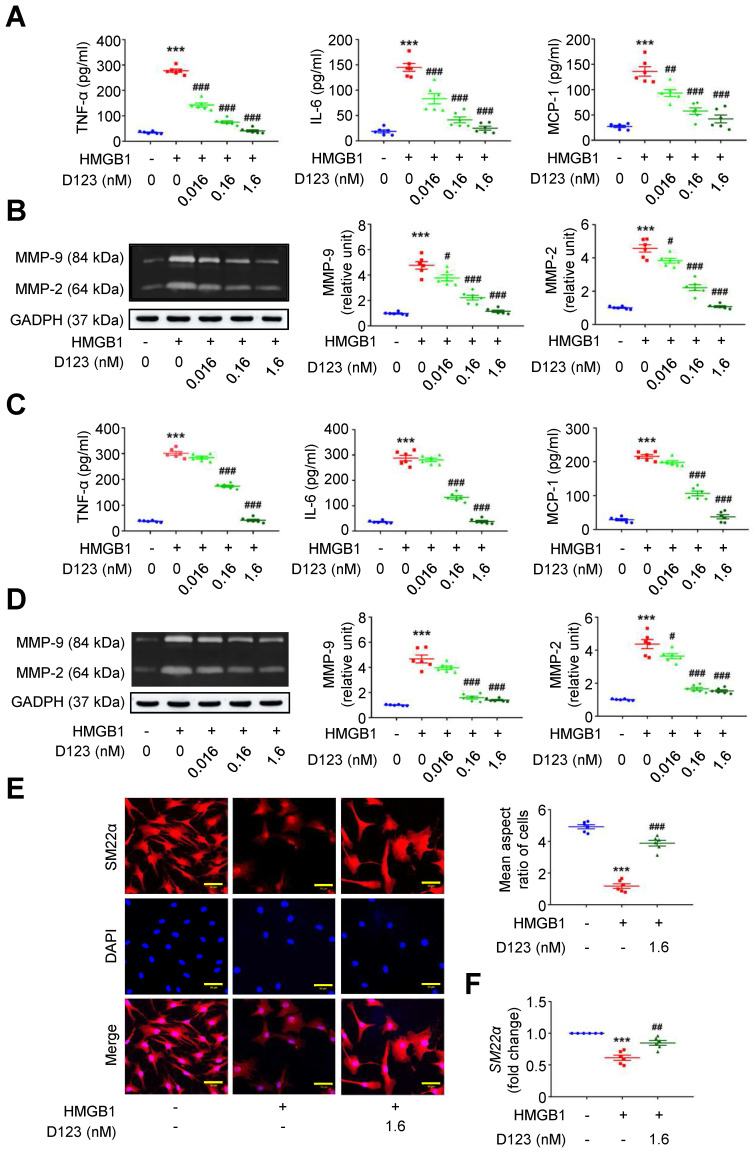
** Soluble rCD93D123 inhibits HMGB1-induced cellular activation in macrophages and VSMCs.** Differentiated THP-1 cells (A, B) and HASMCs (C-E) were treated with HMGB1 (25 nM) and indicated doses of rCD93D123.** (A)** TNF-α, IL-6, and MCP-1 production at 1 day (n = 6). For TNF-α and IL-6, ***P < 0.0001 vs. HMGB1-negative group; ^###^*P <* 0.0001 vs. HMGB1-only group. For MCP-1, ***P < 0.0001 vs. HMGB1-negative group; ^##^*P =* 0.0020 vs. HMGB1-only group; ^###^*P <* 0.0001 vs. HMGB1-only group. **(B)** Representative zymography and quantification of MMP-9 and MMP-2 activities at 1 day (n = 6). For both MMP-9 and MMP-2, ***P < 0.0001 vs. HMGB1-negative group; ^#^*P =* 0.0133 vs. HMGB1-only group; ^###^*P* < 0.0001 vs. HMGB1-only group. **(C)** TNF-α, IL-6, and MCP-1 production at 1 day (n = 6). ***P < 0.0001 vs. HMGB1-negative group; ^###^*P <* 0.0001 vs. HMGB1-only group. **(D)** Representative zymography and quantification of MMP-9 and MMP-2 activities at 1 day (n = 6). For MMP-9, ***P < 0.0001 vs. HMGB1-negative group; ^###^*P <* 0.0001 vs. HMGB1-only group. For MMP-2, ***P < 0.0001 vs. HMGB1-negative group; ^#^*P =* 0.0180 vs. HMGB1-only group; ^###^*P <* 0.0001 vs. HMGB1-only group. **(E)** Representative microscopic images of immunofluorescence staining and quantification of aspect ratio in HASMCs at 1 day (n = 6). ***P < 0.0001 vs. HMGB1-negative group; ^###^*P =* 0.0001 vs. HMGB1-only group. All scale bars represent 50 μm. **(F)** *SM22α* gene expression at 1 days (n = 6). ***P < 0.0001 vs. HMGB1-negative group; ^##^*P =* 0.0076 vs. HMGB1-only group. D123 indicates rCD93D123. Data are represented as mean values ± SEM and comparative statistical analyses were done by one-way ANOVA followed by multiple comparisons.

During AAA formation, inflammatory macrophages and VSMCs are primary sources of proinflammatory cytokines (e.g., TNF-α, interleukin [IL]-6 and monocyte chemoattractant protein [MCP]-1) and extracellular matrix (ECM)-degrading proteinases (e.g., matrix metalloproteinases [MMPs]) 25-27. The HMGB1-receptor interaction activates macrophage and VSMC inflammation that underpins aneurysm growth 23, 28, 29. Given that soluble rCD93 interacts with HMGB1, we explored whether rCD93D123 functionally interferes with cellular activation by HMGB1. HMGB1 enhanced TNF-α secretion in PMA-differentiated THP-1 cells (**Figure 2A**), and this increase was reduced by rCD93D123 treatment in a dose-dependent manner. Similar results were observed in the measurements of IL-6 and MCP-1. As shown by gelatin zymography (**Figure 2B**), HMGB1 increased MMP-9 and MMP-2 activities, and the increases were attenuated by rCD93D123 in a dose-dependent manner. Whether soluble rCD93 hampers the switch of VSMCs to a proinflammatory phenotype by HMGB1 was also explored. HMGB1 increased the production of TNF-α, IL-6, and MCP-1 in human aortic smooth muscle cells (HASMCs; **Figure 2C**), and the increase was dose-dependently inhibited by rCD93D123. Gelatin zymography revealed increased MMP-9 and MMP-2 activities by HMGB1 (**Figure 2D**), and the increase in MMP-9 was attenuated by 0.16 nM and 1.6 nM of rCD93D123, whereas the increase in MMP-2 was attenuated by rCD93D123 in a dose-dependent manner. Immunofluorescence staining revealed that rCD93D123 reversed morphological changes induced by HMGB1 in HASMCs (**Figure 2E**), as quantitatively expressed by the aspect ratio (calculated by dividing the major axis by the minor axis of the bounding ellipse for each cell 30). The differentiated VSMC marker *SM22α* gene expression was suppressed by HMGB1, and the reduction was attenuated by rCD93D123 (**Figure 2F**), suggesting that morphological alterations corresponded to phenotypic properties of VSMCs 31. These findings suggested that soluble rCD93 can not only bind to HMGB1 but also functionally prevent HMGB1-induced cellular activation in macrophages and VSMCs.

### Soluble rCD93, via D1, interferes with HMGB1-induced cellular activation

**Figure 3:**
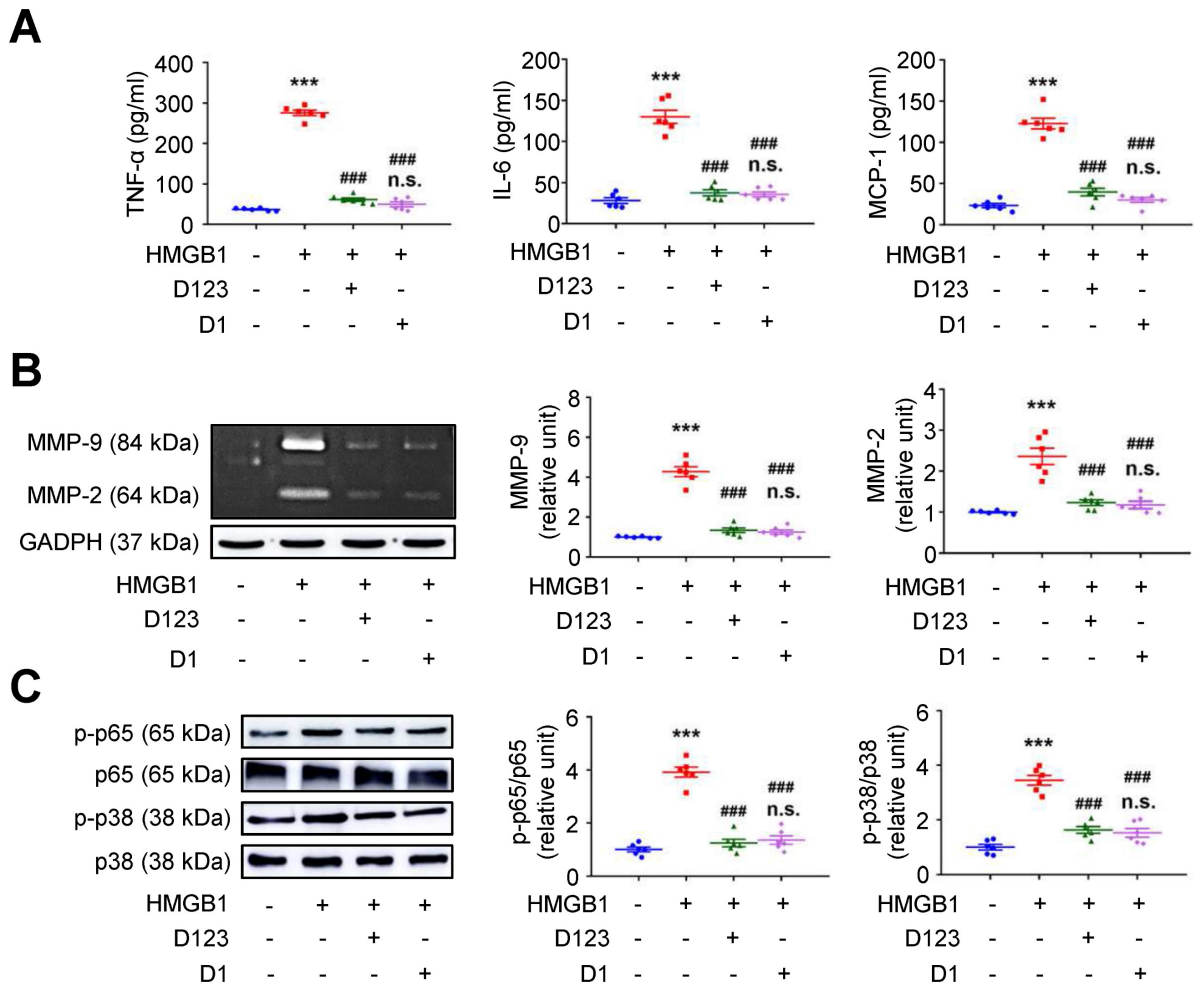
** Soluble rCD93D1 is comparable to rCD93D123 in interfering with HMGB1-induced cellular activation in macrophages.** Differentiated THP-1 cells were treated with HMGB1 (25 nM) and rCD93D123 (1.6 nM) or rCD93D1 (1.6 nM).** (A)** TNF-α, IL-6, and MCP-1 production at 1 day (n = 6). ***P < 0.0001 vs. HMGB1-negative group; ^###^*P* < 0.0001 vs. HMGB1-only group; n.s. *P* > 0.9999 vs. D123-treated group. **(B)** Representative zymography and quantification of MMP-9 and MMP-2 activities at 1 day (n = 6). ***P < 0.0001 vs. HMGB1-negative group; ^###^*P* < 0.0001 vs. HMGB1-only group; n.s. *P* > 0.9999 vs. D123-treated group. **(C)** Representative western blot analysis and quantification of p-p65/p65 and p-p38/p38 levels at 1 hour (n = 6). ***P < 0.0001 vs. HMGB1-negative group; ^###^*P* < 0.0001 vs. HMGB1-only group; n.s. *P* > 0.9999 vs. D123-treated group. D123 indicates rCD93D123. D1 indicates rCD93D1. Data are represented as mean values ± SEM and comparative statistical analyses were done by one-way ANOVA followed by multiple comparisons.

Soluble rCD93 can interact with HMGB1 via D1. Thus, we investigated whether rCD93D1 exerts comparable effects to mole-equivalent rCD93D123 in HMGB1-induced cellular activation. Both rCD93D123 and rCD93D1 inhibited HMGB1-induced increases in TNF-α, IL-6, and MCP-1 secretion (**Figure 3A**) and MMP-9 and MMP-2 activities (**Figure 3B**) in differentiated THP-1 cells, and the levels under rCD93D123 and rCD93D1 were not significantly different.

Analysis of intracellular signaling showed that rCD93D123 and rCD93D1 suppressed HMGB1-induced phosphorylated (p)-p65 NF-κB and p-p38 mitogen-activated protein kinase (MAPK) upregulation (**Figure 3C**), and the expression levels did not significantly differ under rCD93D123 and rCD93D1. In HASMCs, rCD93D123 and rCD93D1 attenuated HMGB1-induced increases in proinflammatory cytokine secretion (**Figure S3A**), MMP activities (**Figure S3B**), and p-p65 NF-κB and p-p38 MAPK signaling (**Figure S3C**), and the levels were similar under rCD93D123 and rCD93D1. The comparable results obtained from rCD93D123 and rCD93D1 treatment indicated that D1 of soluble rCD93 may be the effective domain impeding the proinflammatory effects of HMGB1. Consistent with protein binding studies showing that D1 of soluble rCD93 binds to HMGB1, these observations supported the concept that the proinflammatory effects of HMGB1 may be intercepted in the presence of D1 of soluble rCD93.

### Treatment with soluble rCD93 attenuates AAA *in vivo*

**Figure 4:**
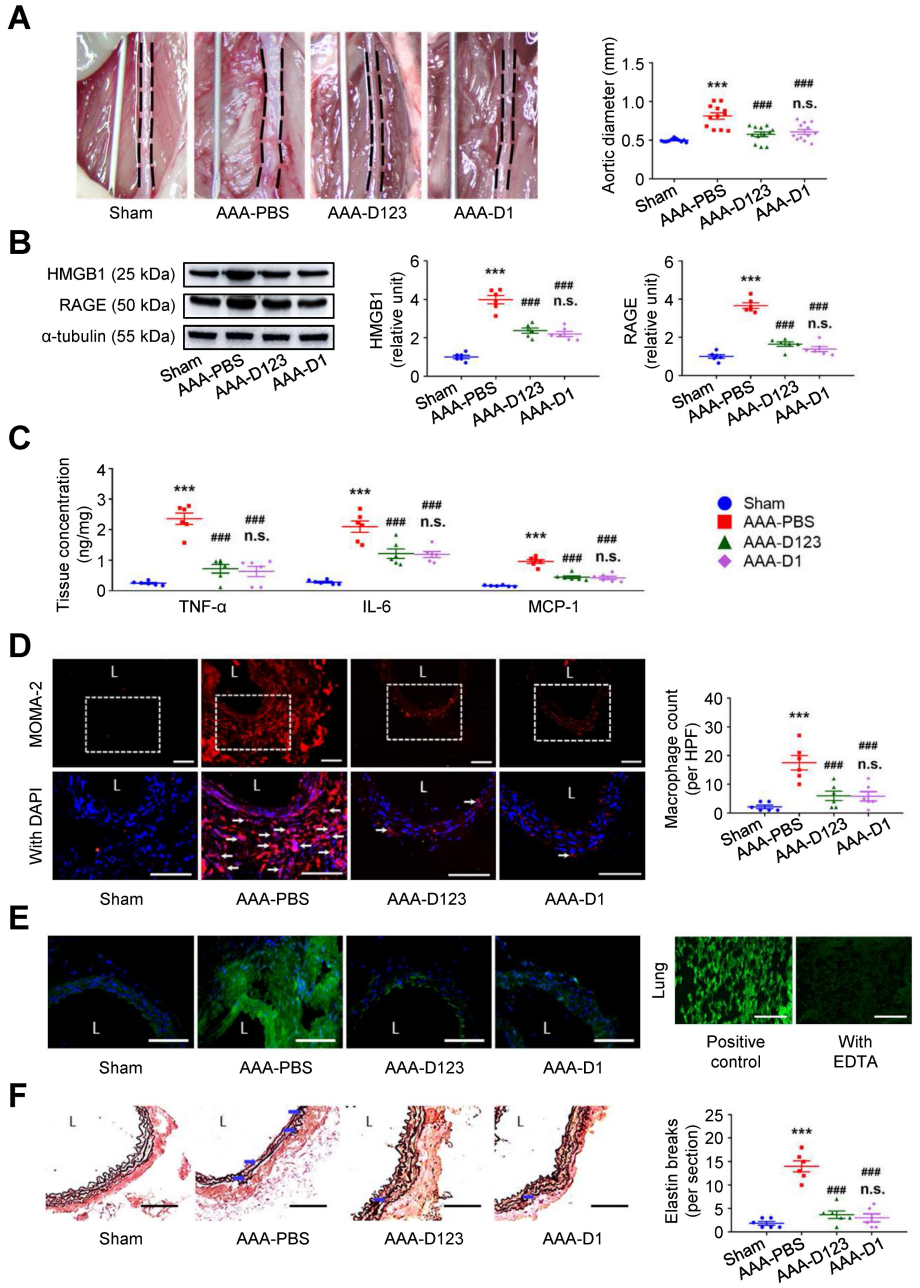
** Treatment with soluble rCD93 attenuates CaCl_2_-induced AAA formation at 4 weeks. (A)** Representative photos of the infrarenal aortas and aortic diameter measurement (n = 12 per group). ***P < 0.0001 vs. sham group; ^###^*P* < 0.0001 vs. AAA-PBS group; n.s. *P* > 0.9999 vs. AAA-D123 group. **(B)** Representative western blot analysis and quantification of HMGB1 and RAGE (n = 6). ***P < 0.0001 vs. sham group; ^###^*P* < 0.0001 vs. AAA-PBS group; n.s. *P* > 0.9999 vs. AAA-D123 group. **(C)** TNF-α, IL-6, and MCP-1 production by ELISA (n = 6). ***P < 0.0001 vs. sham group; ^###^*P* < 0.0001 vs. AAA-PBS group; n.s. *P* > 0.9999 vs. AAA-D123 group. **(D)** Representative microscopic images of immunostaining of MOMA-2-positive macrophages and macrophage count (n = 6). White arrows indicate MOMA-2-positive, DAPI-stained macrophages. ***P < 0.0001 vs. sham group; ^###^*P* < 0.0001 vs. AAA-PBS group; n.s. *P* > 0.9999 vs. AAA-D123 group. **(E)** Representative microscopic images of in situ zymography. The section of lung served as a positive control and the other incubated with EDTA as a negative control. **(F)** Representative microscopic images of VVG staining and elastin break (n = 6). Blue arrows indicate disrupted elastic lamella. ***P < 0.0001 vs. sham group; ^###^*P* < 0.0001 vs. AAA-PBS group; n.s. *P* > 0.9999 vs. AAA-D123 group. D123 indicates rCD93D123. D1 indicates rCD93D1. L indicates lumen. All scale bars represent 50 μm. Data are represented as mean values ± SEM and comparative statistical analyses were done by one-way ANOVA followed by multiple comparisons.

Inhibition of HMGB1-RAGE interaction may suppress AAA formation 23, 28. *In vivo* effects of soluble rCD93 treatment were thus evaluated using two AAA models. In CaCl_2_-induced AAA formation, aortic dilatation was retarded in the rCD93D123-treated mice (0.57 ± 0.03 mm) and the rCD93D1-treated mice (0.60 ± 0.03 mm) compared with the phosphate-buffered saline (PBS)-treated mice (0.81 ± 0.04 mm, both *P* < 0.001; n=12 per group; **Figure 4A**), and the aortic diameter in the rCD93D123-treated mice was not different from that in the rCD93D1-treated mice. HMGB1-RAGE interaction is crucial in maintaining chronic inflammation 18-20, 22. Proinflammatory cytokines (e.g., TNF-α) may activate inflammatory cells (e.g., macrophages) to induce HMGB1 secretion, and in turn, HMGB1 may induce proinflammatory cytokine release in inflammatory cells 18, 19, 32. Analysis of aortic samples revealed that the high abundance of HMGB1 and RAGE (**Figure 4B**) and high levels of TNF-α, IL-6, and MCP-1 (**Figure 4C**) in the PBS-treated mice were substantially reduced by rCD93D123 and rCD93D1 treatment, and the levels in the rCD93D123-treated mice and the rCD93D1-treated mice were not different. Significantly fewer infiltrating monocyte/macrophage marker antibody (MOMA)-2-positive macrophages were found in the rCD93D123-treated mice (6.2 ± 1.8 per high power field [HPF]) and the rCD93D1-treated mice (6.3 ± 1.4 per HPF) than in the PBS-treated mice (17.8 ± 2.5 per HPF; both *P* < 0.01; **Figure 4D**), and the macrophage numbers in these two groups did not differ significantly. As shown by *in situ* zymography, MMP activities were markedly reduced in the rCD93D123-treated and the rCD93D1-treated mice compared with the PBS-treated mice (**Figure 4E**). The histological studies demonstrated loss of the natural waviness accompanied by elastin fragmentation in the PBS-treated mice, and medial elastin integrity was preserved by rCD93D123 and rCD93D1 treatment (**Figure 4F**), compatible with the numbers of elastin breaks. In AngII-infused AAA, both rCD93D123 and rCD93D1 treatment effectively suppressed aortic dilatation (**Figure S4A**) and increases in HMGB1, RAGE (**Figure S4B**), proinflammatory cytokines (**Figure S4C**), infiltrating macrophage numbers (**Figure S4D**), MMP activities (**Figure S4E**), and elastin breaks (**Figure S4F**) induced by AngII infusion. The numbers/levels in the rCD93D123- and rCD93D1-treated mice were not different. The comparable effectiveness of rCD93D123 and rCD93D1 treatment revealed that D1 of soluble rCD93 may be the effective domain in suppressing AAA formation. These *in vitro and in vivo* findings suggested that treatment with soluble rCD93 containing D1 can attenuate inflammation and proteolysis in AAA, at least in part, through inhibition of HMGB1-RAGE signaling.

### HMGB1 potentiates osteoclastogenesis stimulated by RANKL (receptor activator of NF-κB ligand)

Next, we investigated the effect of soluble rCD93 on osteoclastogenesis, an essential process during bone metabolism that can be promoted by inflammatory signals 16, 33-35. During osteoclastogenesis, RANKL induces precursor monocyte/macrophage fusion and differentiation into multinuclear osteoclasts responsible for bone resorption 36. Evaluating the subcellular distribution of HMGB1 in RAW264.7 macrophages revealed that RANKL stimulated HMGB1 translocation from the nucleus to the cytoplasmic compartment and release into the culture medium in a dose-dependent manner (**Figure S5A**). Immunofluorescence staining showed that RANKL stimulation substantially accentuated the staining of HMGB1 that was colocalized with F-actin (**Figure S5B**), a prerequisite cytoplasmic structure for osteoclast bone resorption 34. Thus, RANKL may stimulate HMGB1 translocation and release in macrophages during osteoclastogenesis.

**Figure 5:**
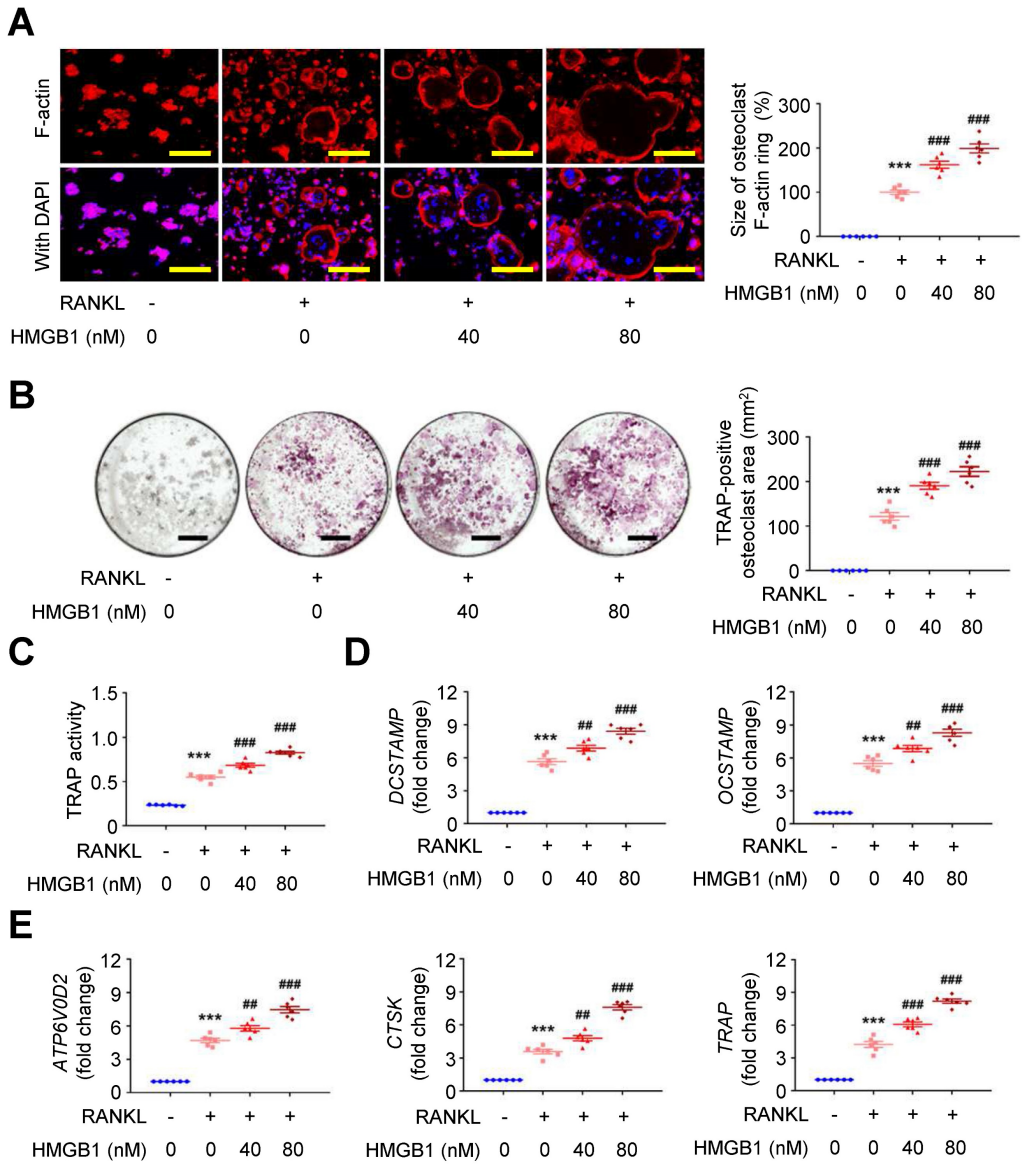
** HMGB1 potentiates RANKL-stimulated osteoclastogenesis.** RAW264.7 macrophages were treated with RANKL (30 ng/ml) and indicated doses of HMGB1. **(A)** Representative microscopic images of immunofluorescence staining of F-actin and quantification of F-actin size at 4 days (n = 6). ***P < 0.0001 vs. RANKL-negative group; ^###^*P* < 0.0001 vs. RANKL-only group. **(B)** Representative microscopic images of TRAP staining and quantification of TRAP-positive osteoclast area at 4 days (n = 6). ***P < 0.0001 vs. RANKL-negative group; ^###^*P* < 0.0001 vs. RANKL-only group. **(C)** TRAP activity at 4 days (n = 6). ***P < 0.0001 vs. RANKL-negative group; ^###^*P* < 0.0001 vs. RANKL-only group. **(D)** *DCSTAMP* and* OCSTAMP* gene expression at 2 days (n = 6). For *DCSTAMP*, ***P < 0.0001 vs. RANKL-negative group; ^##^*P* = 0.0063 vs. RANKL-only group; ^###^*P* < 0.0001 vs. RANKL-only group. For *OCSTAMP*, ***P < 0.0001 vs. RANKL-negative group; ^##^*P* = 0.0061 vs. RANKL-only group; ^###^*P* < 0.0001 vs. RANKL-only group. **(E)** *ATP6V0D2*, *CTSK*, and *TRAP* gene expression at 2 days (n = 6). For *ATP6V0D2*, ***P < 0.0001 vs. RANKL-negative group; ^##^*P* = 0.0095 vs. RANKL-only group; ^###^*P* < 0.0001 vs. RANKL-only group. For *CTSK*, ***P < 0.0001 vs. RANKL-negative group; ^##^*P* = 0.0020 vs. RANKL-only group; ^###^*P* < 0.0001 vs. RANKL-only group. For *TRAP*, ***P < 0.0001 vs. RANKL-negative group; ^###^*P* < 0.0001 vs. RANKL-only group. Yellow scale bars represent 100 μm. Black scale bars represent 1 mm. Data are represented as mean values ± SEM and comparative statistical analyses were done by one-way ANOVA followed by multiple comparisons.

Exploring the effect of HMGB1 in the presence of RANKL on RAW264.7 macrophages using immunofluorescence staining showed that RANKL-induced F-actin ring formation was dose-dependently expanded by HMGB1 (**Figure 5A**). Additionally, the area positive for the osteoclast marker tartrate-resistant acid phosphatase (TRAP; **Figure 5B**) and TRAP activity (**Figure 5C**) in RAW264.7 macrophages were increased by RANKL, and these effects were additionally enhanced by HMGB1 in a dose-dependent manner. The mRNA expression levels of osteoclast fusion genes, *DCSTAMP* and* OCSTAMP* (**Figure 5D**) 37, and osteoclast differentiation marker genes, *ATP6V0D2*,* CTSK*, and* TRAP* (**Figure 5E**), were induced by RANKL. These gene expression levels were enhanced by HMGB1. These findings suggested that HMGB1 may be actively secreted under RANKL stimulation and in return promote RANKL-induced osteoclastogenesis, consistent with previous studies identifying HMGB1 as an osteoclastogenic cytokine 34.

### Soluble rCD93, via D1, inhibits HMGB1-RAGE signaling and impedes osteoclastogenesis

**Figure 6:**
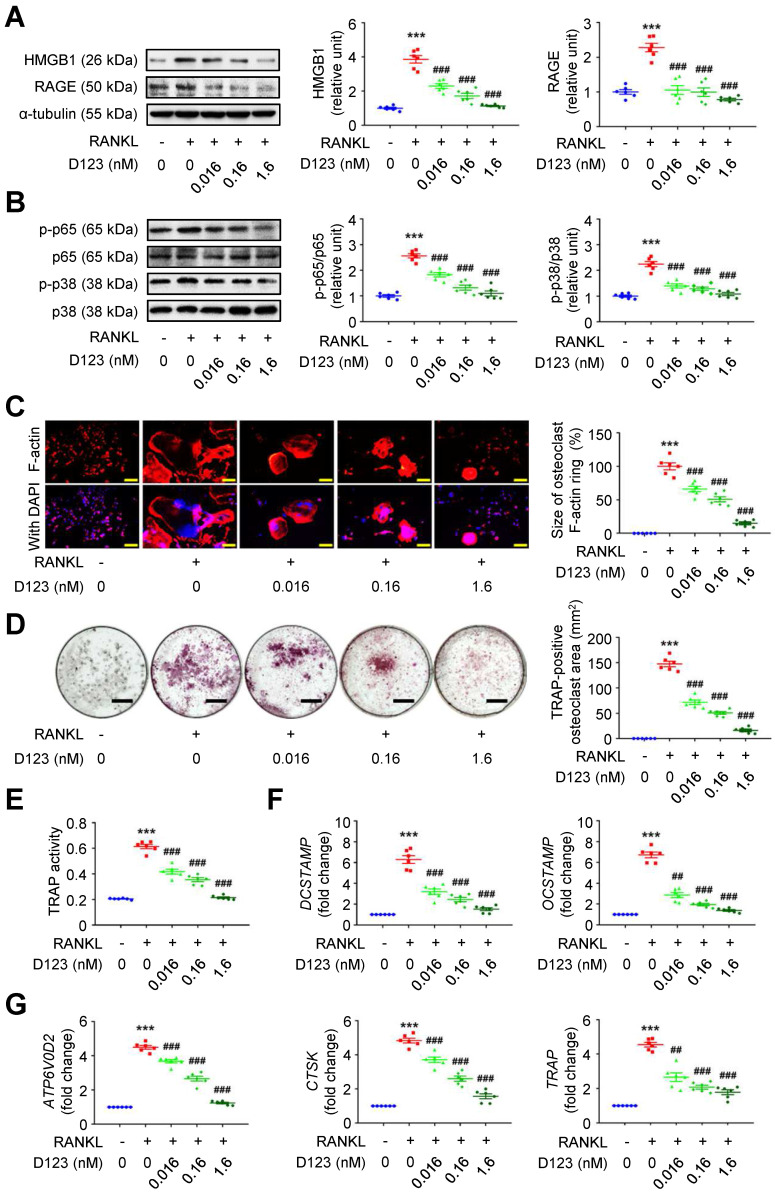
** Soluble rCD93D123 suppresses osteoclastogenesis.** RAW264.7 macrophages were treated with RANKL (30 ng/ml) and indicated doses of rCD93D123. **(A)** Representative western blot analysis and quantification of HMGB1 and RAGE levels at 1 day (n = 6). ***P < 0.0001 vs. RANKL-negative group. ^###^*P* < 0.0001 vs. RANKL-only group. **(B)** Representative western blot analysis and quantification of p-p65/p65 and p-p38/p38 levels at 1 hour (n = 6). ***P < 0.0001 vs. RANKL-negative group; ^###^*P* < 0.0001 vs. RANKL-only group. **(C)** Representative microscopic images of immunofluorescence staining of F-actin and quantification of F-actin size at 4 days (n = 6). ***P < 0.0001 vs. RANKL-negative group; ^###^*P* < 0.0001 vs. RANKL-only group. **(D)** Representative microscopic images of TRAP staining and quantification of TRAP-positive osteoclast area at 4 days (n = 6). ***P < 0.0001 vs. RANKL-negative group; ^###^*P* < 0.0001 vs. RANKL-only group. **(E)** TRAP activity at 4 days (n = 6). ***P < 0.0001 vs. RANKL-negative group; ^###^*P* < 0.0001 vs. RANKL-only group. **(F)** *DCSTAMP* and* OCSTAMP* gene expression at 2 days (n = 6). For *DCSTAMP,* ***P < 0.0001 vs. RANKL-negative group; ^###^*P* < 0.0001 vs. RANKL-only group. For *OCSTAMP*, ***P < 0.0001 vs. RANKL-negative group; ^##^*P* = 0.0022 vs. RANKL-only group; ^###^*P* < 0.0001 vs. RANKL-only group. **(G)** *ATP6V0D2*, *CTSK*, and *TRAP* gene expression at 2 days (n = 6). For *ATP6V0D2* and* CTSK,* ***P < 0.0001 vs. RANKL-negative group; ^###^*P* < 0.0001 vs. RANKL-only group. For *TRAP*, ***P < 0.0001 vs. RANKL-negative group; ^##^*P* = 0.0034 vs. RANKL-only group; ^###^*P* < 0.0001 vs. RANKL-only group. Yellow scale bars represent 200 μm. Black scale bars represent 1 mm. D123 indicates rCD93D123. Data are represented as mean values ± SEM and comparative statistical analyses were done by one-way ANOVA followed by multiple comparisons.

Given that soluble rCD93 can bind to HMGB1, we investigated whether soluble rCD93 functionally impedes osteoclastogenesis, during which HMGB1 is a potent driving factor. RAW264.7 macrophages were treated with RANKL and different concentrations of rCD93D123. The proinflammatory HMGB1 signaling amplification sustains autocrine or paracrine positive feedback loops 18-20, 22. Analysis of signaling pathways involved in osteoclastogenesis revealed that RANKL upregulated HMGB1 and its surface receptor RAGE (**Figure 6A**), p-p65 NF-κB and p-p38 MAPK pathways (**Figure 6B**), and the upregulation was dose-dependently attenuated by rCD93D123. The sizes of RANKL-induced F-actin ring formation were reduced dose-dependently by rCD93D123 (**Figure 6C**). As shown by the TRAP-positive osteoclast area (**Figure 6D**) and TRAP activity (**Figure 6E**), RANKL-induced osteoclast differentiation in RAW264.7 macrophages was inhibited by rCD93D123 in a dose-dependent manner. The gene expression levels of RANKL-induced osteoclastogenic marker genes, including* DCSTAMP*,* OCSTAMP* (**Figure 6F**),* ATP6V0D2*,* CTSK*, and* TRAP* (**Figure 6G**), were dose-dependently attenuated by rCD93D123. Soluble rCD93 can impede RANKL-induced osteoclastogenesis at least partly through inhibiting HMGB1-RAGE interaction and downstream cascades.

**Figure 7:**
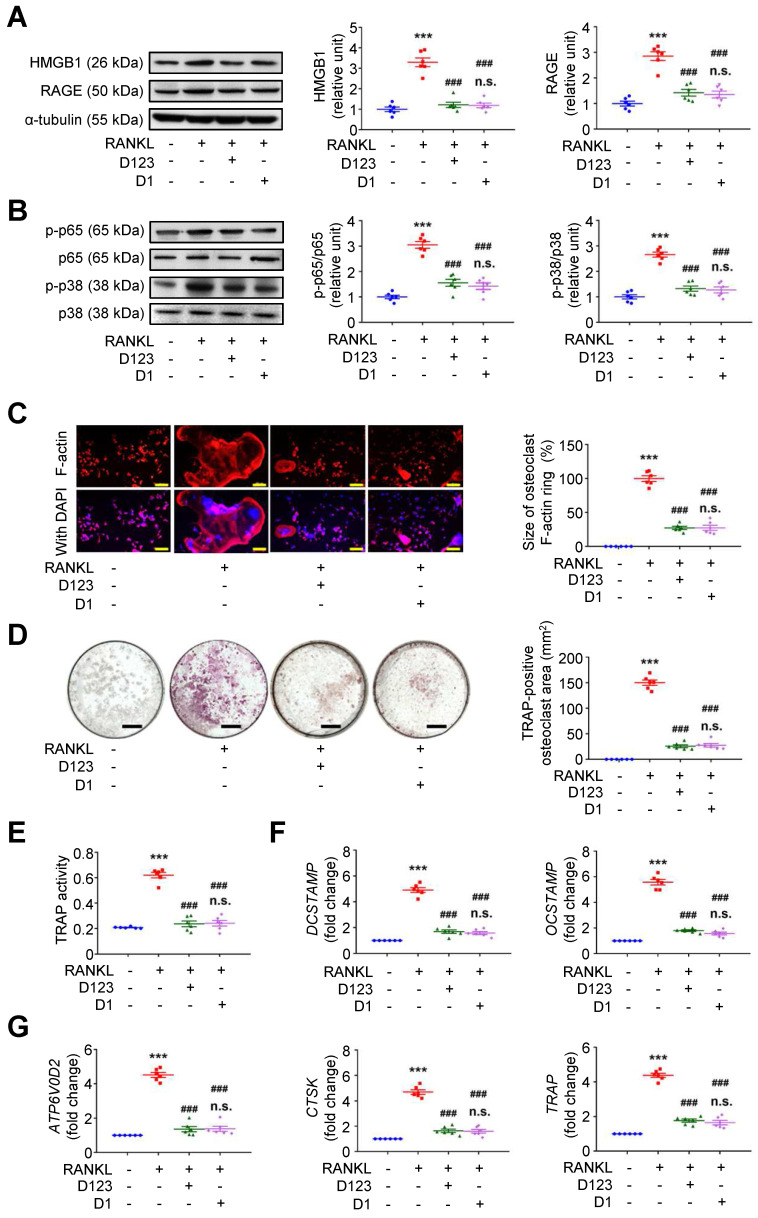
** Soluble rCD93D1 is comparable to rCD93D123 in hindrance of osteoclastogenesis.** RAW264.7 macrophages were treated with RANKL (30 ng/ml) and rCD93D123 (1.6 nM) or rCD93D1 (1.6 nM). **(A)** Representative western blot analysis and quantification of HMGB1 and RAGE levels at 1 day (n = 6). ***P < 0.0001 vs. RANKL-negative group; ^###^*P* < 0.0001 vs. RANKL-only group; n.s. *P* > 0.9999 vs. D123-treated group.** (B)** Representative western blot analysis and quantification of p-p65/p65 and p-p38/p38 levels at 1 hour (n = 6). ***P < 0.0001 vs. RANKL-negative group; ^###^*P* < 0.0001 vs. RANKL-only group; n.s. *P* > 0.9999 vs. D123-treated group. **(C)** Representative microscopic images of immunofluorescence staining of F-actin and quantification of F-actin size at 4 days (n = 6). ***P < 0.0001 vs. RANKL-negative group; ^###^*P* < 0.0001 vs. RANKL-only group; n.s. *P* > 0.9999 vs. D123-treated group. **(D)** Representative microscopic images of TRAP staining and quantification of TRAP-positive osteoclast area at 4 days (n = 6). ***P < 0.0001 vs. RANKL-negative group; ^###^*P* < 0.0001 vs. RANKL-only group; n.s. *P* > 0.9999 vs. D123-treated group. **(D)** TRAP activity at 4 days (n = 6). * ***P < 0.0001 vs. RANKL-negative group; ^###^*P* < 0.0001 vs. RANKL-only group; n.s. *P* > 0.9999 vs. D123-treated group. **(E)** *DCSTAMP* and* OCSTAMP* gene expression at 2 days (n = 6). ***P < 0.0001 vs. RANKL-negative group; ^###^*P* < 0.0001 vs. RANKL-only group; n.s. *P* > 0.9999 vs. D123-treated group. **(F)** *ATP6V0D2*, *CTSK*, and *TRAP* gene expression at 2 days (n = 6). ***P < 0.0001 vs. RANKL-negative group; ^###^*P* < 0.0001 vs. RANKL-only group; n.s. *P* > 0.9999 vs. D123-treated group. Yellow scale bars represent 200 μm. Black scale bars represent 1 mm. D123 indicates rCD93D123. D1 indicates rCD93D1. Data are represented as mean values ± SEM and comparative statistical analyses were done by one-way ANOVA followed by multiple comparisons.

Because soluble rCD93 can interact with HMGB1 via D1, we investigated whether rCD93D1 exerts comparable effects to mole-equivalent rCD93D123 in osteoclastogenesis. In RAW264.7 macrophages, RANKL-induced upregulation of HMGB1 and RAGE (**Figure 7A**), p-p65 NF-κB and p-p38 MAPK pathways (**Figure 7B**) was suppressed by rCD93D123 and rCD93D1 treatment, and the expression levels were not different under rCD93D123 and rCD93D1. The sizes of RANKL-induced F-actin ring formation were reduced by rCD93D123 and rCD93D1, and the sizes were not different under rCD93D123 and rCD93D1 (**Figure 7C**). RANKL-induced increases in the TRAP-positive osteoclast area (**Figure 7D**) and TRAP activity (**Figure 7E**) were inhibited by rCD93D123 and rCD93D1, and these levels were not different under rCD93D123 and rCD93D1. Also, RANKL-induced upregulation of *DCSTAMP*,* OCSTAMP* (**Figure 7F**),* ATP6V0D2*,* CTSK*, and* TRAP* (**Figure 7G**) gene expression was attenuated by rCD93D123 and rCD93D1, and the levels were not different under rCD93D123 and rCD93D1. The comparable results from rCD93D123 and rCD93D1 treatment suggested that D1 of soluble rCD93 is likely the active domain hindering the pro-osteoclastogenic effects of HMGB1, compatible with protein binding studies. On the basis of these data, we speculated that treatment with soluble rCD93 containing D1 might potentially attenuate bone resorption through interference with HMGB1-RAGE interaction, which crucially contributes to osteoclastogenesis and bone resorption 33, 34.

### Treatment with soluble rCD93 alleviates OVX-induced osteoporosis* in vivo*

**Figure 8:**
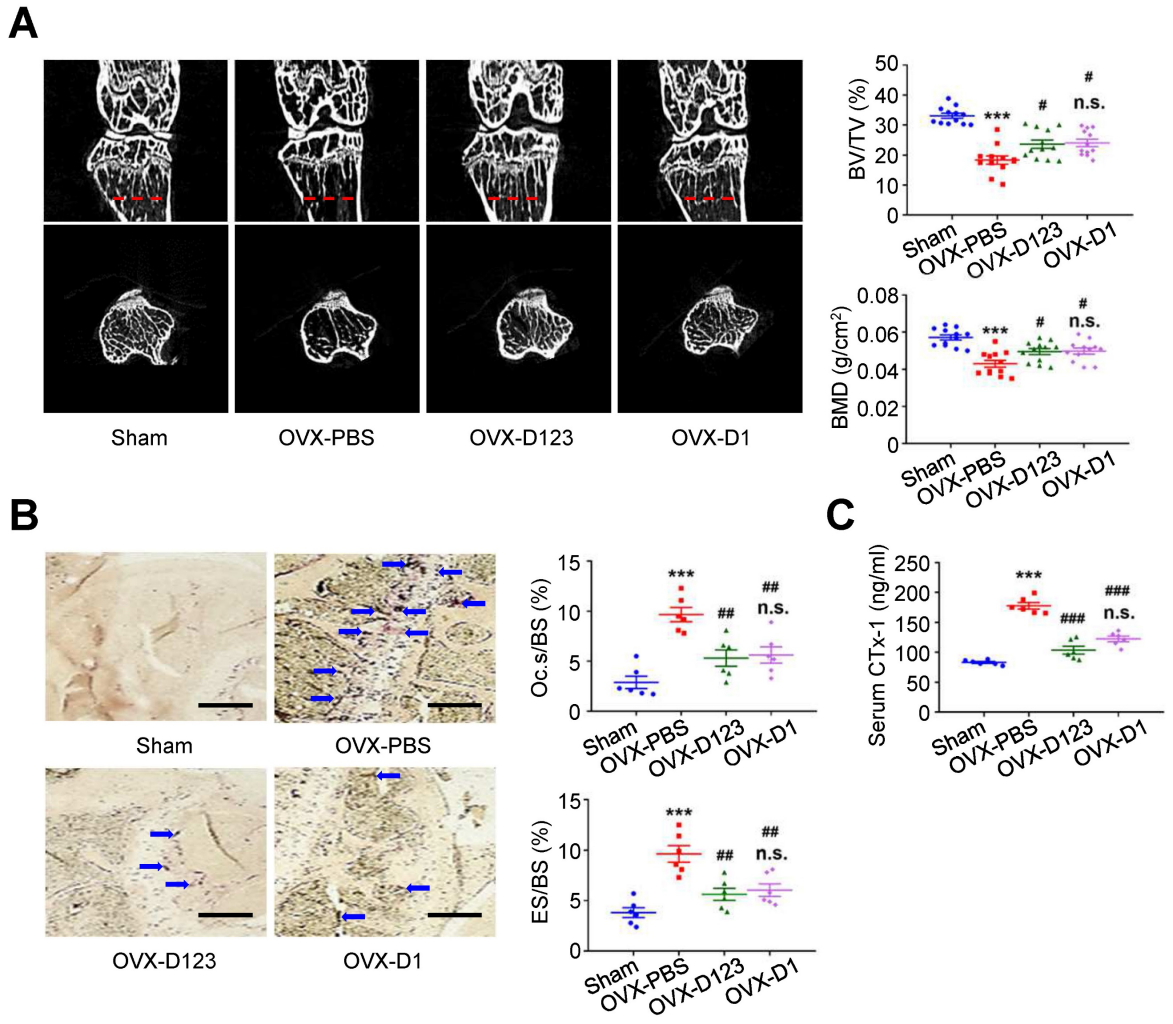
** Treatment with soluble rCD93 alleviates OVX-induced osteoporosis at 8 weeks. (A)** Representative μ-CT scanning images of the trabecular bones and quantification of trabecular bone volume/total bone volume (BV/TV) and bone mineral density (BMD; n = 12). For BV/TV, ***P < 0.0001 vs. sham group; ^#^*P* = 0.0224 OVX-D123 vs. OVX-PBS group; ^#^*P* = 0.0113 OVX-D1 vs. OVX-PBS group; n.s. *P* > 0.9999 vs. OVX-D123 group. For BMD, ***P < 0.0001 vs. sham group; ^#^*P* = 0.0378 OVX-D123 vs. OVX-PBS group; ^#^*P* = 0.0282 OVX-D1 vs. OVX-PBS group; n.s. *P* > 0.9999 vs. OVX-D123 group. **(B)** Representative microscopic images of TRAP-stained histological sections of the trabecular bone and quantification of osteoclast surface/bone surface (Oc.s/BS) and eroded surface/bone surface (ES/BS). For Oc.s/BS, ***P < 0.0001 vs. sham group; ^##^*P* = 0.0031 OVX-D123 vs. OVX-PBS group; ^##^*P* = 0.0061 OVX-D1 vs. OVX-PBS group; n.s; *P* > 0.9999 vs. OVX-D123 group. For ES/BS, ***P < 0.0001 vs. sham group; ^##^*P* = 0.0017 OVX-D123 vs. OVX-PBS group; ^##^*P* = 0.0066 OVX-D1 vs. OVX-PBS group; n.s; *P* > 0.9999 vs. OVX-D123 group. **(C)** Serum CTx-1 (n = 6). ***P < 0.0001 vs. sham group; ^###^*P* < 0.0001 vs. OVX-PBS group; n.s. *P* > 0.0843 vs. OVX-D123 group. Dashed lines indicate the cross sections. Blue arrows indicate TRAP-positive osteoclasts. All scale bars represent 50 μm. D123 indicates rCD93D123. D1 indicates rCD93D1. Data are represented as mean values ± SEM and comparative statistical analyses were done by one-way ANOVA followed by multiple comparisons.

Finally, the effect of rCD93D123 and rCD93D1 treatment on *in vivo* bone loss was evaluated using the OVX-induced model. At 8 weeks, micro-computed tomography (μ-CT) scans showed that the reduced trabecular bone volume fraction (i.e., trabecular bone volume [BV]/ total bone volume [TV]) and bone mineral density (BMD) in the PBS-treated mice were rescued in the rCD93D123- and rCD93D1-treated mice (**Figure 8A**), and the levels in the rCD93D123- and rCD93D1-treated mice were not different. TRAP staining on histological sections showed that the increased ratios of osteoclast-covered surface (Oc.s) and eroded surface (ES) relative to the total bone surface (BS) in the PBS-treated mice were alleviated in the rCD93D123- and rCD93D1-treated mice (**Figure 8B**), and the ratios in the rCD93D123- and rCD93D1-treated mice were not different. Consistent with the histological findings, the increased serum level of the bone resorption marker C-terminal telopeptide of type I collagen (CTx-1) 38 in the PBS-treated mice was reduced in the rCD93D123- and rCD93D1-treated mice (**Figure 8C**), and the levels in the rCD93D123- and rCD93D1-treated mice were not different. Taken together, the *in vitro and in vivo* findings suggested that treatment with soluble rCD93 containing D1 attenuated osteoclastogenesis and bone resorption at least partly through inhibiting HMGB1-receptor interaction.

## Discussion

Through protein binding studies, we find that soluble rCD93 can bind to HMGB1 to prevent HMGB1 engagement with its cell receptor RAGE, and this interaction occurs at the level of D1. Accordingly, we evaluate the effect of soluble rCD93 containing D1 using a variety of *in vitro* and* in vivo* inflammatory responses, including macrophage and VSMC activation, CaCl_2_-induced and AngII-infused AAA formation, RANKL-induced osteoclastogenesis, and OVX-induced osteoporosis. Soluble rCD93 containing D1 ameliorates experimental AAA formation, possibly through inhibition of HMGB1-induced macrophage and VSMC activation. Also, soluble rCD93 containing D1 attenuates bone resorption, possibly through inhibition of HMGB1-enhanced osteoclastogenesis. Current knowledge regarding whether sCD93 exhibits proinflammatory or anti-inflammatory activity comes from *in vitro* studies, but their findings are conflicting. One study showed that soluble rCD93 induces the differentiation of monocytes to macrophage-like cells and enhances toll-like receptor (TLR) responses to lipopolysaccharide stimulation 39. More recently, however, another group observed that soluble rCD93D1 mitigates lipopolysaccharide-induced proinflammatory responses 40. Based on the observations obtained from different cell culture experiments and disease models, the present study demonstrates the therapeutic potential of soluble rCD93 containing D1 in inflammatory diseases and provides evidence to support the anti-inflammatory property of sCD93. In addition, the anti-inflammatory mechanism identified in the present study is quite distinct from the well-known function of sCD93 in efferocytosis.

The significance of HMGB1-RAGE signaling has been established in the mechanisms of sepsis, lung injury, arthritis, and other acute and chronic inflammatory diseases 16, 19, 22. The proinflammatory HMGB1 may contribute through its cell receptors, together with other DAMPs 16, 18-22, to a biologic amplification mechanism to drive inflammatory disorders such as aneurysm formation and bone resorption. Studies have demonstrated the roles of HMGB1 and its receptors RAGE and TLR4 in the pathogenesis of AAA 23, 24, 28, 29. HMGB1 neutralization using therapeutic antibody reduces proinflammatory cytokine production, attenuates macrophage infiltration and suppresses CaCl_2_-induced AAA formation in mice 28. RAGE deficiency prevents aortic dilatation in AngII-infused AAA formation with eliminated MMP-9 production in macrophages 24. During aneurysm formation, HMGB1 may sustain inflammation through RAGE and TLR4 23, 29, leading to the upregulation of proinflammatory cytokines and proteinases that degrade ECM. Previous studies have also revealed the essential role of HMGB1-RAGE interaction during osteoclastogenesis 16, 33, 34. RAGE-deficient mice have decreased bone resorptive activity and increased bone mass and mineral density 33. As shown in our study, RANKL stimulated HMGB1 secretion during osteoclastogenesis. HMGB1 positively regulates RANKL-induced osteoclastogenesis in a manner dependent on RAGE 34, and sustained RAGE activation plays a vital role in chronic cellular activation and tissue damage 18-20, 22. RANKL collaborates reciprocally with HMGB1-RAGE signaling in inflammatory bone loss. HMGB1-RAGE interaction plays a critical role in the development of AAA and osteoporosis. Previously, extensive studies have demonstrated that the proinflammatory HMGB1 signaling sustains positive feedback loops through an autocrine or paracrine manner 18-20, 22. During AAA formation, the inflammatory responses mediated by HMGB1-RAGE interaction can be mitigated by soluble rCD93 containing D1, as evidenced by the reduced abundance of HMGB1, RAGE, and other inflammatory mediators. These findings are consistent with protein binding and *in vitro* studies showing that soluble rCD93 containing D1 can not only intercept HMGB1-RAGE interaction but also functionally inhibit HMGB1-induced macrophage and VSMC activation. In addition, the inflammatory bone loss associated with HMGB1-RAGE interaction can be alleviated by soluble rCD93 containing D1, as reflected by the reduction of HMGB1 and RAGE abundance and osteoclastogenic marker gene expression during osteoclastogenesis. These findings, together with protein binding study results, are in line with the observations in AAA and further corroborate the concept that soluble rCD93 containing D1 sequesters HMGB1 to ameliorate inflammatory diseases.

Interfering with HMGB1-receptor interaction by HMGB1 sequestration is proposed as a promising strategy to alleviate HMGB1-related inflammatory disorders 16, 21. As demonstrated in the present study, soluble rCD93 ectodomain proteins containing D1 can bind to HMGB1. The therapeutic benefits of rCD93D123 and rCD93D1 in HMGB1-driven inflammatory diseases suggest that soluble rCD93 containing D1 acts like a decoy receptor. Such a strategy can also be accomplished by several established approaches 23, 28, 34, 35, such as HMGB1 neutralizing antibody, sRAGE, and recombinant thrombomodulin (CD141) containing the lectin-like domain.

Among the C‐type lectin-like domain group 14 family members, including thrombomodulin, tumor endothelial marker 1 (TEM1; CD248, also called endosialin), and C-type lectin domain containing 14A, the amino acid alignment of human CD93 is most closely related to that of thrombomodulin 2, 40. It is speculated that CD93 and thrombomodulin are derived from a common ancestor due to their strong homology and close proximity on chromosome 20 2, 3, 40, 41. The lectin-like domain of soluble form thrombomodulin can regulate inflammation by sequestering HMGB1 and lipopolysaccharide 17, 42. Through these actions, soluble recombinant thrombomodulin containing the lectin-like domain exerts therapeutic benefits in a variety of inflammatory disease models *in vivo* 17, 35, 42-44. In the present study, the observations from *in vitro* and *in vivo* experiments suggest the anti-inflammatory function of soluble rCD93 containing D1, the lectin-like domain. These findings provide evidence to support the long-standing speculation of functional similarity in modulating inflammation between CD93 and thrombomodulin 2. Correspondingly, given that membrane-bound thrombomodulin (i.e., thrombomodulin expression on the cell surface) in macrophages exerts a proinflammatory function that is opposite to the general understanding regarding the anti-inflammatory activity of soluble thrombomodulin 45, 46, the role of membrane-bound CD93 during inflammation needs to be reappraised. Interestingly, current understanding regarding the function of membrane-bound CD93 in inflammatory responses is also contradictory 47-50. CD93 deficiency leads to increased inflammation and severity in experimentally-induced cerebral ischemia and encephalomyelitis 48, 49, consistent with the finding that CD93-deficient mice have increased leukocyte recruitment during thioglycollate-induced peritonitis 47. However, a recent study demonstrated that membrane-bound CD93 might act as a cell surface receptor for exogenous DNA and mediate downstream inflammation in a CD93-expressing IMR-32 neuroblastoma cell line 50, implying a proinflammatory property of membrane-bound CD93. Further studies are warranted to explore whether membrane-bound CD93 exerts a proinflammatory or anti-inflammatory function in inflammatory responses.

## Conclusions

In conclusion, the present study demonstrates an anti-inflammatory property of sCD93/rCD93 residing in the lectin-like domain. These findings provide insight by demonstrating a novel anti-inflammatory mechanism, i.e., sequestering HMGB1 by sCD93 via the lectin-like domain, distinct from the function of sCD93 in efferocytosis. Links between sCD93 containing the lectin-like domain and the protective effect against HMGB1 suggest that sCD93-HMGB1 interaction may be relevant to *in vitro* and* in vivo* inflammatory responses and that the soluble rCD93 lectin-like domain holds promise in potential therapies for AAA and osteoporosis.

## Methods

**Expression and purification of soluble rCD93 ectodomain proteins.** The soluble rCD93 ectodomain proteins with a c-Myc epitope, including rCD93D123 (Thr22-Lys580), rCD93D1 (Thr22-Gly177), and rCD93D23 (Val258-Lys580), were prepared as described previously 4. In brief, the HEK293 cell protein expression system was used to express soluble rCD93 ectodomain proteins purified using a nickel-chelating Sepharose column (Amersham Pharmacia Biotech, Piscataway, NJ). The purified rCD93D123, rCD93D1, and rCD93D23 proteins were evaluated by sodium dodecyl sulfate-polyacrylamide gel electrophoresis (SDS-PAGE) with Coomassie blue staining and analyzed by immunoblotting using anti-c-Myc antibody (sc-40, Santa Cruz Biotechnology, Santa Cruz, CA; 1:1000).

**Assays for rCD93-HMGB1 interaction.** For co-immunoprecipitation (Co-IP) assay, rCD93D123 (5 nM) and HMGB1 (5 nM; R&D Systems, Minneapolis, MN) were incubated for 1 hour at 37 °C in immunoprecipitation buffer (50 mM Tris-HCl, 100 mM NaCl, 0.25% Triton X-100, 1 mM MgCl_2_, and 2 mM EGTA; final volume: 0.5 ml). Protein A agarose (20 μl; Millipore, Billerica, MA) conjugated with a rabbit polyclonal antibody against HMGB1 (1 μg; ab18256, Abcam, Cambridge, MA) or normal IgG, serving as a negative control, was added to the protein mixtures (200 μl) and then incubated overnight at 4 °C. Protein A agarose was collected by centrifugation and washed. The immune precipitate was solubilized in sample buffer (10 μl). Samples were loaded to 10% SDS-PAGE followed by a western blot assay with anti-c-Myc antibody (sc-40, Santa Cruz Biotechnology; 1:1000). Subsequently, the membranes were incubated with the appropriate secondary antibodies at room temperature for 1 hour. The immunoreactive bands were detected by chemiluminescence reagents (Millipore). Far-western blot assay was conducted as the following. rCD93D1, rCD93D23, rCD93D123, and bovine serum albumin (BSA; 0.1 μg/ml) were separated by 10% SDS-PAGE, transferred to a polyvinylidene difluoride membrane, and then hybridized with HMGB1 (0.5 μg/ml) in PBS overnight at 4 °C. After appropriate wash steps, the membranes were incubated with rabbit anti-HMGB1 antibody (ab18256, Abcam; 1:1000) overnight at 4 °C, followed by an HRP-conjugated secondary antibody. Ponceau S staining (Sigma-Aldrich, St Louis, MO), serving as the loading control, was performed based on the manufacturer's protocols. ELISA solid-phase binding assay was performed as the following. Ninety-six-well ELISA plates with high protein-binding capacity (Nunc, Roskilde, Denmark) were coated with indicated doses of rCD93D123, rCD93D1, rCD93D23, and BSA overnight at 4 °C with coating buffer (0.15% Na_2_CO_3_, 1% MgCl_2_, and 0.3% NaHCO_3_ in double-distilled water). The wells were washed with phosphate-buffered saline with Tween 20 (PBST) and then incubated with blocking buffer (50 mg/ml BSA, 20 mM Tris-HCl, 0.15 M NaCl, and 5 mM CaCl_2_ in double-distilled water) for 2 hours at room temperature. After PBST washes, indicated doses of HMGB1 were incubated with reaction buffer (1 mg/ml BSA, 20 mM Tris-HCl, 0.15 M NaCl, and 5 mM CaCl_2_ in double-distilled H_2_O) overnight at 4 °C. The competitive ELISA assay was performed as the following. Ninety-six-well ELISA plates were coated with sRAGE (10 nM; Sino Biological, Beijing, China) overnight at 4 °C with a coating buffer. After being blocked with a blocking buffer and washed with PBST, the indicated concentration of rCD93D123, rCD93D1, rCD93D23, HMGB1, and BSA were incubated in the sRAGE-coated plate with reaction buffer overnight at 4 °C. The ELISA plates were washed and then incubated with anti-HMGB1 antibody (ab18256, Abcam; 1:1000) for 2 hours at room temperature, followed by an HRP-conjugated antibody (GeneTex, Irvine, CA) for 2 hours at room temperature. Tetramethylbenzidine (Sigma-Aldrich) was applied as a substrate and incubated for 20 minutes, followed by the addition of 50 μl of 1 M H_2_SO_4_ per well to stop the reaction. The absorbance at 450 nm was measured in a microplate reader (DYNEX Technologies, Denkendorf, Germany). For flow cytometry assay, live or fixed RAW264.7 and CHO cells were incubated with HMGB1 (20 nM) in the presence of sRAGE, rCD93D123, rCD93D1, or rCD93D23 (10 nM each) for 1 hour at 37 °C. Cells were stained with anti-HMGB1 antibody (ab18256, Abcam; 1:100) overnight at 4 °C followed by Alexa Fluor 546-conjugated rabbit IgG (Invitrogen, Carlsbad, CA; 1:500) for 30 minutes at room temperature and analyzed by fluorescence activated cell sorting (FACS; BD Biosciences, San Jose, CA). The histogram was generated using WinMDI 2.9 software (Purdue University cytometry laboratories, West Lafayette, IN).

Regarding potential contamination in the HMGB1 preparation, according to the datasheet, the lipopolysaccharide level was less than 0.1 EU per 1 μg of the protein measured by the Limulus amoebocyte lysate assay. The amount of DNA in HMGB1 (100 ng) was measured using a NanoDrop 2000 spectrophotometer (Thermo Fisher Scientific, Waltham, MA, USA) with a negative control (Milli-Q ultrapure water) and a positive control (genomic DNA isolated from the mouse tail). The results suggested the absence of DNA in the HMGB1 preparation (**Table S1**).

***In vitro* cell culture.** Human monocytic THP-1 cells were obtained from the Bioresource Collection and Research Center in Taiwan. Cells were maintained in an RPMI medium with 10% FBS, 1 mM L-glutamine, sodium pyruvate, and penicillin/streptomycin in a 5% CO_2_ incubator at 37 °C and were plated in a medium containing 10 nM phorbol-12-myristate-13-acetate (PMA; Sigma-Aldrich) for 18 hours for differentiation. HASMCs (Cascade Biologics, Portland, OR) were cultured in Medium 231 containing 10% FBS, 1% smooth muscle growth supplement, and penicillin/streptomycin in a 5% CO_2_ incubator at 37 °C. Mouse RAW264.7 cells (Bioresource Collection and Research Center) were cultured in α-MEM with 1% FBS, 2 mM L-glutamine, and penicillin/streptomycin within a 5% CO_2_ incubator at 37 °C. In addition to, RAW264.7 cells were stimulated with macrophage-colony stimulating factor (20 ng/ml) and indicated doses of RANKL for osteoclast differentiation. The culture medium was replenished every 2 days. THP-1 cells, HASMCs, and RAW264.7 cells were treated with HMGB1, rCD93D123, and rCD93D1 as indicated in the experiments.

**AAA models and soluble rCD93 treatment.** The CaCl_2_-induced and AngII-infused AAA mouse models were established as previously described 44, 51. These mice were maintained in a pathogen-free animal facility at the Animal Center of National Cheng Kung University. Only male mice were used due to the potential influence of female sex hormones on AAA models and the male predominance of human AAAs 52, 53. For CaCl_2_-induced AAA, the male C57BL/6J mice (The Jackson Laboratory, Bar Harbor, ME) aged 9-12 weeks were used. The CaCl_2_-induced AAA model was induced with 0.5 M CaCl_2_ applied onto the infrarenal aorta for 15 minutes under anesthesia. For AngII-infused AAA, the male ApoE^-/-^ mice (The Jackson Laboratory) aged 6 months were used. The AngII-infused AAA model was generated using the continuous subcutaneous infusion of AngII (1000 ng/kg/min; Sigma-Aldrich) via osmotic pumps (Alzet 2004; Durect, Cupertino, CA). To evaluate the effectiveness of soluble rCD93 treatment in AAA models, mice were treated with rCD93D123 (intraperitoneal injection with rCD93D123 [320 μg/kg] in 0.2 mL PBS once a day every 3 days starting the day after CaCl_2_ injury or pump implantation), a mole-equivalent dosage of rCD93D1 (intraperitoneal injection with rCD93D1 [104 μg/kg] in 0.2 mL PBS once a day every 3 days starting the day after CaCl_2_ injury or pump implantation), or the same volume of PBS (n=12 per group for CaCl_2_-induced AAA; n=8 per group for AngII-infused AAA). At 4 weeks, the mice were sacrificed for aortic harvest. For the CaCl_2_-induced AAA model, the segment from the left renal artery to the aortic bifurcation was harvested, and for the AngII-infused AAA model, the section between the last pair of intercostal arteries and the right renal branch was harvested for analysis. Different aortic segments were harvested for analysis because CaCl_2_-induced AAA was induced by the direct application of CaCl_2_ onto the infrarenal aorta, whereas AngII-infused AAA was observed in the suprarenal aortic area. The microscopic image was acquired post-mortem with a 26-gauge needle (0.45 mm in diameter) as a reference to measure the aortic diameter before the aortic sample was resected. The images were processed using ImageJ software (National Institutes of Health, Bethesda, MD), and the aortic diameter was determined in proportion to the needle diameter. The maximal aortic diameter in each mouse was blindly determined by 2 independent observers, and the data were then averaged. The value for each mouse in each group is presented. These experiments were approved by the Institutional Animal Care and Use Committee of National Cheng Kung University (approval number: 108092) and conformed to the *Guide for the Care and Use of Laboratory Animals* published by the National Institutes of Health (NIH Publication #85-23, revised 1996).

**OVX-induced osteoporosis model and soluble rCD93 treatment.** Female C57BL/6J mice (The Jackson Laboratory, Bar Harbor, ME) aged 8 weeks were used and maintained in a pathogen-free animal facility at Kaohsiung Medical University Animal Center. The OVX-induced bone loss model was generated by bilateral ovariectomy under anesthesia 54. To evaluate the effectiveness of soluble rCD93 treatment in OVX-induced bone loss, rCD93D123 (intraperitoneal injection with rCD93D123 [0.6 mg/kg] in 0.1 mL PBS once a day every 3 days since the following day after OVX), a mole-equivalent dosage of rCD93D1 (intraperitoneal injection with rCD93D1 [0.2 mg/kg] in 0.1 mL PBS once a day every 3 days since the following day after OVX), or the same volume of PBS (n = 12 per group) was used. At 8 weeks, the mice were sacrificed for μ-CT evaluation and lower limb harvest. All μ-CT analyses were performed in accordance with the guidelines 55. Bone samples from each group were imaged using a SkyScan-1076 μ-CT System (Skyscan, Kontich, Belgium). The μ-CT scanner was operated at 45 kV, 220 μA, 0.4 μ rotation step, 0.5 mm aluminum filter, and a scan resolution of 18 μm/pixel. The following 3D parameters, including BMD, TV, BV, and BV/TV, were evaluated using CT Analyzer software (Bruker, Kontich, Belgium). These experiments were approved by the Animal Care and Use Committee of Kaohsiung Medical University Animal Center (approval number: 110004) and conformed to the *Guide for the Care and Use of Laboratory Animals* published by the National Institutes of Health (NIH Publication #85-23, revised 1996).

***In vitro* immunofluorescence assay.** After stimulation, HASMCs and RAW264.7 cells were washed with PBS and fixed with 4% paraformaldehyde at room temperature for 30 minutes, followed by permeabilization with 0.1% Triton X-100 for 5 minutes at 4 °C. After being blocked with PBS containing 10% BSA for 1 hour at room temperature, cells were incubated with anti-SM22α (ab14106, Abcam; 1:500) or anti-HMGB1 (ab18256, Abcam; 1:500) antibodies overnight at 4 °C. Cells were washed and incubated with fluorescent-dye conjugated secondary antibodies (Invitrogen) at room temperature for 1 hour, washed using PBS, and then stained with 4', 6-diamidino-2-phenylindole (DAPI) for 5 minutes. The fluorescence was visualized using a confocal laser scanning microscope coupled with an image analysis system (Olympus Fluoview FV1000, Olympus, Tokyo, Japan). For the morphological changes of HASMCs, 3 random view-fields were used to measure the aspect ratio of individual cells. The aspect ratio was calculated through the division of the major axis by the minor axis of the bounding ellipse for each cell using Imaris software (Andor Technology, Belfast, Northern Ireland) 30, 56.

**ELISA quantification.** Human TNF-α (DY210, R&D Systems), human IL-6 (DY206, R&D Systems), and human MCP-1 (DY279, R&D Systems) in culture media, mouse TNF-α (DY410, R&D Systems), mouse IL-6 (DY406, R&D Systems), and mouse MCP-1 (DY479, R&D Systems) in mouse abdominal aortic homogenates and mouse CTx-1 (CEA665Mu, Cloud-Clone, Katy, TX) in the serum were analyzed using commercially available ELISA kits based on the manufacturers' protocols.

**Western blot analysis and gelatin zymography.** Cells were harvested using ice-cold lysis buffer after being washed twice with ice-cold PBS. The protein homogenate from cells or aortic samples was centrifuged at 12000 rpm for 25 minutes at 4 °C, and the supernatant was recovered as the total cellular protein. Separation and preparation of cytoplasmic and nuclear extracts were performed using the NE-PER Nuclear and Cytoplasmic Extraction Reagent Kit (Pierce Biotechnology, Rockford, IL) as needed according to the manufacturer's instructions. The protein from each sample was separated by SDS-PAGE on 12% acrylamide gels, transferred to a PVDF membrane, and then blocked with 5% non-fat dry milk in Tris-buffered saline with Tween 20. The membrane was incubated with primary antibodies in PBST at 4 °C overnight. The primary antibodies used in the study were antibodies against human and mouse p-p65 (#3033, Cell Signaling, Danvers, MA; 1:1000), p65 (sc-8008, Santa Cruz; 1:1000), p-p38 (#4511, Cell Signaling; 1:1000), p38 (#9212, Cell Signaling; 1:1000), HMGB1 (ab18256, Abcam; 1:1000), RAGE (A13264, ABclonal, Woburn, MA,; 1:1000), GAPDH (GTX627408, GeneTex; 1:1000), α-tubulin (GTX112141, GeneTex; 1:1000), and lamin B2 (33-2100, Thermo Fisher, Waltham, MA; 1:1000). Subsequently, the membranes were incubated with appropriate secondary antibodies at room temperature for 1 hour, followed by chemiluminescence reagents (Millipore). MMP-2 and MMP-9 activities in the supernatants obtained from the culture medium were demonstrated using gelatin zymography. The supernatant was separated using 7.5% SDS-PAGE containing 0.1% gelatin. After incubation with the activation buffer overnight at 37 °C, SDS-PAGE was performed with Coomassie blue staining. The band intensity in western blot analysis and gelatin zymography was quantitatively measured using ImageJ software. GADPH, α-tubulin and lamin B2 levels were determined to confirm equal protein loading.

**TRAP activity, TRAP staining, and F-actin ring formation in cell culture.** TRAP activity was measured using 4-nitrophenyl phosphate (4-NPP) as a substrate 35. A 50-μl cell lysate was incubated with 100 μl of substrate solution (0.1 M 4-NPP, 0.1 M sodium acetate, and 0.2 M sodium tartrate, pH 5.0) for 60 minutes at 37 °C. The reaction was terminated by adding 50 μl of 3 M NaOH. Absorbance was measured at 405 nm in a microplate reader (DYNEX Technologies). For TRAP staining, cells were fixed and stained with a TRAP staining kit (387A, Sigma-Aldrich). TRAP-positive multinucleated cells with 3 or more nuclei were counted as osteoclasts. The TRAP-positive osteoclast area per well was quantified using AxioVision software (Carl Zeiss, Oberkochen, Germany). To observe F-actin ring formation, cells were fixed with 4% paraformaldehyde for 30 minutes and permeabilized with 0.1% Triton X-100 for 5 minutes. After being blocked with PBS containing 10% BSA for 1 hour, cells were incubated with rhodamine-conjugated phalloidin (Invitrogen; 1:1000) in PBS for 1 hour at room temperature, followed by washing using PBS and DAPI staining. The fluorescence images were captured using a confocal laser scanning microscope coupled with an image analysis system (Olympus Fluoview FV1000). In each well, 3 random view-fields were counted to quantify the size of the F-actin ring using AxioVision software.

**Real-time quantitative reverse transcription PCR.** Total RNA was extracted from cells using the SV Total RNA Isolation System (Promega, Madison, WI) 54. Based on the manufacturer's instructions, first-strand cDNA was synthesized by the Reverse Transcription System Kit (Promega) with 500 ng of total RNA. The PCR mixture was prepared using 40 ng cDNA, SYBR Green Low Rox quantitative PCR mixes, and 0.5 μM primers. The sequences of primers are listed as follows: human *SM22α* (forward, 5'-TCC AGA CTG TTG ACC TCT TTG-3' and reverse, 5'-TCT TAT GCT CCT GCG CTTC-3') and *HPRT* (forward, 5'-TCT TTG CTG ACC TGC TGG ATT ACA-3' and reverse, 5'-AGT TGA GAG ATC ATC TCC ACC AAT-3'), mouse* DCSTAMP* (forward, 5'-GGT CTC CTA GAC AGC ATG ACT-3' and reverse, 5'-GCA AGG CCG TAA ATC CAC TG-3'),* OCSTAMP* (forward, 5'-TTG CTC CTG TCC TAC AGT GC-3' and reverse, 5'-GCC CTC AGT AAC ACA GCT CA-3'), *ATP6V0D2* (forward, 5'-TTC TTG AGT TTG AGG CCG AC-3' and reverse, 5'-CAG CTT GAG CTA ACA ACC GC-3'), *CTSK* (forward, 5'-GCG TTG TTC TTA TTC CGA GC-3' and reverse, 5'-CAG CAG AGG TGT GTA CTA TG-3'), *TRAP* (forward, 5'-GAT GCC AGC GAC AAG AGG TT-3' and reverse, 5'-CAT ACC AGG GGA TGT TGC GAA-3'), and *GAPDH* (forward, 5'-AGG TCA TCC CTG AGC TGA ACGG-3' and reverse, 5'-CGC CTG CTT CAC CAC CTT CTTG-3'). Real-time PCR was performed on the ABI StepOnePlus System (Applied Biosystems, Foster City, CA) using Power SYBR Green PCR Master Mix (Takara Bio, Otsu, Japan). The expression levels were normalized with *HPRT* or *GAPDH* as an internal control using the 2^-∆∆Ct^ method.

**Histological analysis.** Frozen sections (5 μm thick) of aortic samples at the maximal aortic dilatation of infrarenal aortas (CaCl_2_-induced AAA) or suprarenal aortas (AngII-infused AAA) were used in staining or immunostaining for MOMA-2 (Abcam; 1:200) and elastin degradation (Verhoeff-Van Gieson [VVG] staining, Abcam). For each mouse, the macrophage numbers and elastin breaks of 5 serial sections were evaluated blindly by 2 independent observers. *In situ* zymography was performed using elastin conjugated with quenched fluorescein (DQ-gelatin; Invitrogen) as a substrate, which requires cleavage by elastinolytic enzymes to become fluorescent 46. Unfixed aortic cryosections were incubated in the presence of 40 mg/mL DQ-gelatin at 37 °C for one hour. Lung sections served as a positive control, and those incubated with EDTA as a negative control. The fluorescent signal was captured using a fluorescence microscope (DP73; Olympus, Tokyo, Japan). Extracted lower limbs from the OVX-induced osteoporosis model were fixed in 10% formalin, and complete decalcification of the bone samples was conducted using 10% EDTA for 14 days. The lower limbs were sectioned (5 μm thick) in the coronal plane through the central weight-bearing region of the anterior and posterior femorotibial joint, i.e., parallel to the axis of the lower limb. Three sections representing the central weight-bearing area of each femorotibial joint were stained with TRAP staining kit (387A, Sigma-Aldrich). Three random view fields were analyzed per section to quantify Oc.s/BS and ES/BS using AxioVision software.

**Statistics analysis.** All data were expressed as mean ± SEM. One-way analysis of variance followed by post hoc analysis (Bonferroni test) was used in data that passed both normality and equal variance (Bartlett's test); otherwise, a non-parametric Kruskal-Wallis test followed by Dunn's post hoc analysis was used. Statistical analyses were performed using Prism 6 (GraphPad Software, San Diego, CA) and a *P* < 0.05 was considered statistically significant.

## Supplementary Material

Supplementary figures and table.

## Abbreviations

sCD93: soluble CD93
HMGB1: high-mobility group box 1
DAMP: damage-associated molecular pattern
TNF: tumor necrosis factor
RAGE: receptor for advanced glycation end-product
NF: nuclear factor
rCD93: recombinant human CD93
AngII: angiotensin II
AAA: abdominal aortic aneurysm
OVX: ovariectomy
ELISA: enzyme-linked immunosorbent assay
sRAGE: soluble RAGE
CHO: Chinese hamster ovary
IL-6: interleukin-6
MCP-1: monocyte chemoattractant protein-1
ECM: extracellular matrix
MMP: matrix metalloproteinase
HASMC: human aortic smooth muscle cell
MAPK: mitogen-activated protein kinase
PBS: phosphate-buffered saline
MOMA: monocyte/macrophage marker antibody
HPF: high power field
RANKL: receptor activator of nuclear factor κB ligand
TRAP: tartrate-resistant acid phosphatase
μ-CT: micro-computed tomography
BV: trabecular bone volume
TV: total bone volume
BMD: bone mineral density
Oc.s: osteoclast-covered surface
ES: eroded surface
BS: total bone surface
CTx-1: C-terminal telopeptide of type I collagen
TLR: toll-like receptor
Co-IP: co-immunoprecipitation assay
BSA: bovine serum albumin
VVG: Verhoeff-Van Gieson
