# Supplementary material for: Soluble CD93 lectin-like domain sequesters HMGB1 to ameliorate inflammatory diseases
Source: Theranostics. 2023 Jul 14;13(12):4059–78. doi: 10.7150/thno.84935 (PMC10405849; doi:10.7150/thno.84935)
Supplement: Supplementary file 1 — Supplementary figures and table. [file thnov13p4059s1.pdf]

DNA (ng/μl)	Milli-Q ultrapure water (negative control)	Genomic DNA isolated from the mouse tail (positive control)	HMGB1 (100 ng)
1st test	-1.3	137.3	-0.4
2nd test	-0.2	178.5	-0.4
3rd test	-0.7	189.0	-0.2

Table S1. Readings from the spectrophotometer for Milli-Q ultrapure water (negative control), genomic DNA isolated from the mouse tail (positive control), and HMGB1. The readings showed that DNA could be detected in neither Milli-Q water nor HMGB1, indicating the absence of DNA in the HMGB1 preparation.

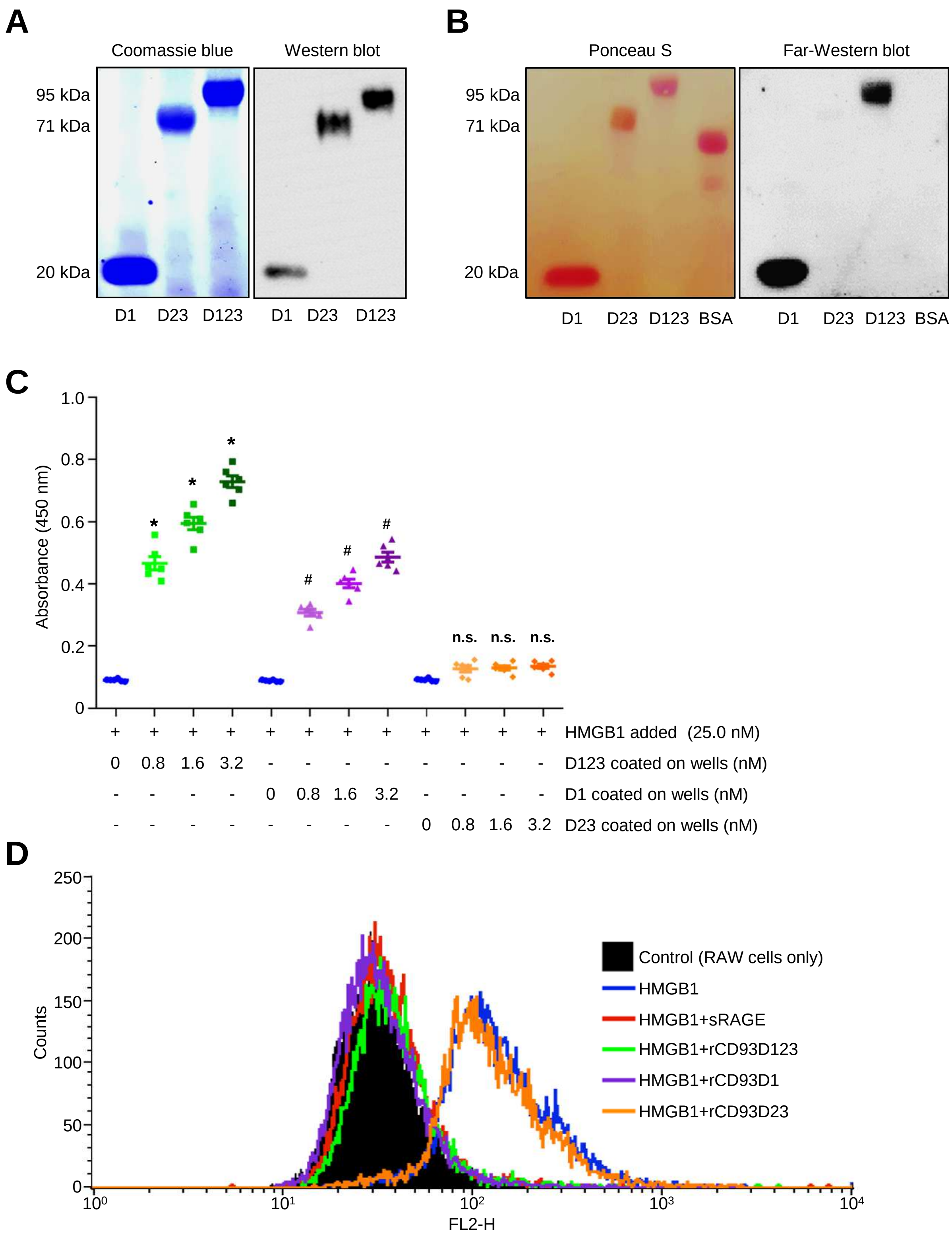

Figure S1. Soluble rCD93D123 and rCD93D1 bind to HMGB1 and prevent HMGB1 engagement with its receptor RAGE. (A) The three purified soluble rCD93 ectodomain proteins were analyzed using Coomassie blue staining and western blot analysis with anti-c-Myc antibody. Representative results from three independent experiments are shown. **(B)** The protein binding between HMGB1 and soluble rCD93 ectodomain proteins or BSA (negative control) was examined using far-western blot analysis. Ponceau S staining serves as the loading control. Representative results from three independent experiments are shown. **(C)** The protein binding between HMGB1 and soluble rCD93 ectodomain proteins was examined using ELISA solid-phase binding assay (n = 6). **P* = 0.0007 HMGB1-rCD93D123 (0.8 nM) vs. HMGB1-rCD93D123 (0 nM) binding; **P* = 0.0001 HMGB1-rCD93D123 (1.6 nM) vs. HMGB1-rCD93D123 (0 nM) binding; **P* < 0.0001 HMGB1-rCD93D123 (3.2 nM) vs. HMGB1-rCD93D123 (0 nM) binding; #*P* = 0.0003 HMGB1-rCD93D1 (0.8 nM) vs. HMGB1-rCD93D1 (0 nM) binding; #*P* = 0.0001 HMGB1-rCD93D1 (1.6, 3.2 nM) vs. HMGB1-rCD93D1 (0 nM) binding; n.s. *P* > 0.9999 HMGB1-rCD93D23 (0.8 nM) vs. HMGB1-rCD93D23 (0 nM) binding; n.s. *P* = 0.1436 HMGB1-rCD93D23 (1.6 nM) vs. HMGB1-rCD93D23 (0 nM) binding; n.s. *P* = 0.0906 HMGB1-rCD93D23 (3.2 nM) vs. HMGB1-rCD93D23 (0 nM) binding. **(D)** The competitive inhibition on HMGB1 binding to RAW264.7 cells by soluble rCD93 ectodomain proteins was examined using flow cytometry. Representative results from three independent experiments are shown. D123, D1, and D23 indicate rCD93D123, rCD93D1, and rCD93D23, respectively. Data are represented as mean values ± SEM and comparative statistical analyses were done by one-way ANOVA followed by multiple comparisons.

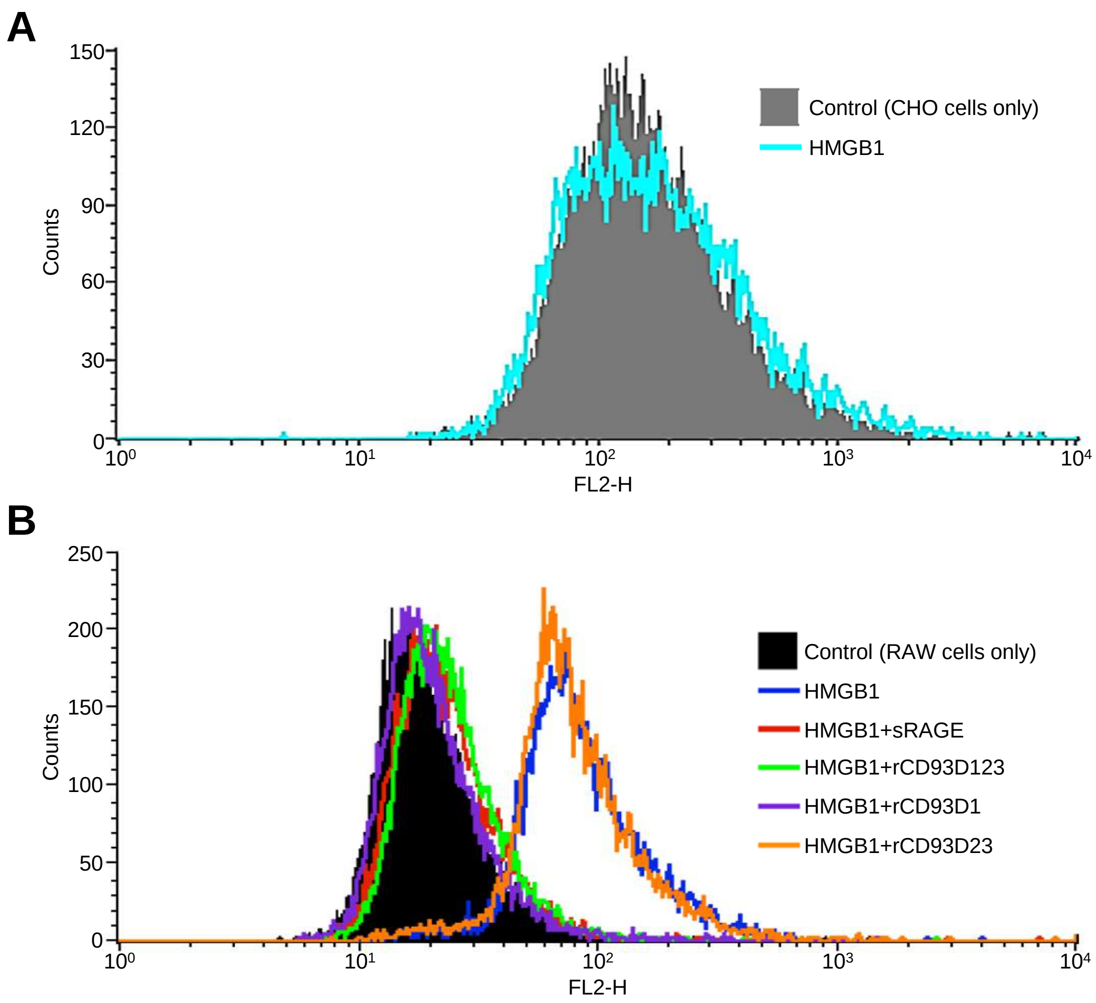

Figure S2. Soluble rCD93D123 and rCD93D1 inhibit HMGB1-macrophage interaction on fixed macrophages. (A) The binding of HMGB1 to fixed CHO cells was examined using flow cytometry. **(B)** The competitive inhibition on HMGB1 binding to fixed RAW264.7 cells by soluble rCD93 ectodomain proteins was examined using flow cytometry. Representative results from three independent experiments are shown.

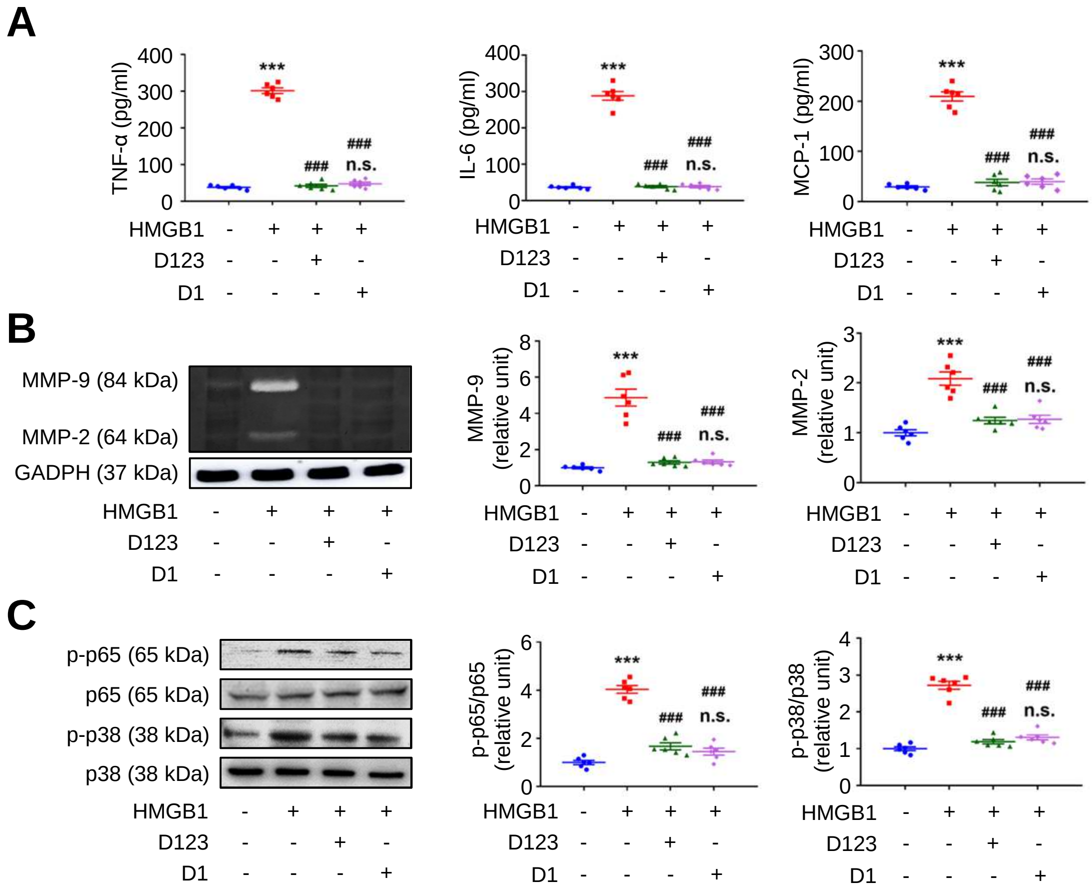

Figure S3. Soluble rCD93D1 is comparable to rCD93D123 in interference with HMGB1-induced cellular activation in VSMCs. HASMCs were treated with HMGB1 (25 nM) and rCD93D123 (1.6 nM) or rCD93D1 (1.6 nM). **(A)** TNF-α, IL-6, and MCP-1 production at 1 day (n = 6). ****P* < 0.0001 vs. HMGB1-negative group; ###*P* < 0.0001 vs. HMGB1-only group; n.s. *P* > 0.9999 vs. D123-treated group. **(B)** Representative zymography and quantification of MMP-9 and MMP-2 activities at 1 day (n = 6). ****P* < 0.0001 vs. HMGB1-negative group; ###*P* < 0.0001 vs. HMGB1-only group; n.s. *P* > 0.9999 vs. D123-treated group. **(C)** Representative western blot analysis and quantification of p-p65/p65 and p-p38/p38 levels at 1 hour (n = 6). ****P* < 0.0001 vs. HMGB1-negative group; ###*P* < 0.0001 vs. HMGB1-only group; n.s. *P* > 0.9999 vs. D123-treated group. D123 indicates rCD93D123. D1 indicates rCD93D1. Data are represented as mean values ± SEM and comparative statistical analyses were done by one-way ANOVA followed by multiple comparisons.

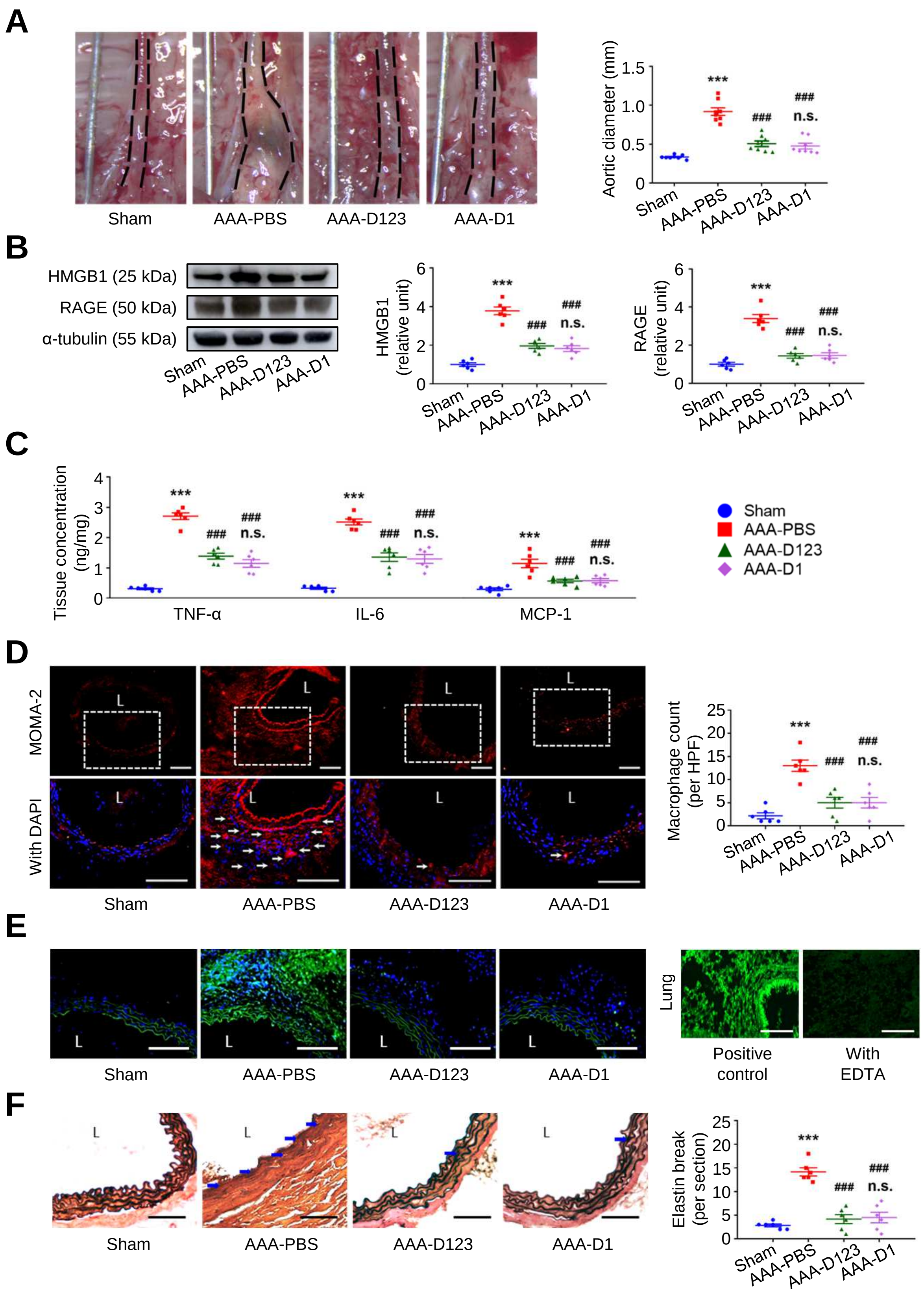

Figure S4. Treatment with soluble rCD93 suppresses AngII-infused AAA at 4 weeks. (A) Representative photos of the suprarenal aortas and aortic diameter measurement (n = 8 per group). ****P* < 0.0001 vs. sham group; ###*P* < 0.0001 vs. AAA-PBS group; n.s. *P* > 0.9999 vs. AAA-D123 group. **(B)** Representative western blot analysis and quantification of HMGB1 and RAGE (n = 6). ****P* < 0.0001 vs. sham group; ###*P* < 0.0001 vs. AAA-PBS group; n.s. *P* > 0.9999 vs. AAA-D123 group. **(C)** TNF-α, IL-6, and MCP-1 production by ELISA (n = 6). For TNF-α, ****P* < 0.0001 vs. sham group; ###*P* < 0.0001 vs. AAA-PBS group; n.s. *P* > 0.9977 vs. AAA-D123 group. For IL-6 and MCP-1, ****P* < 0.0001 vs. sham group; ###*P* < 0.0001 vs. AAA-PBS group; n.s. *P* > 0.9999 vs. AAA-D123 group. **(D)** Representative microscopic images of immunostaining of MOMA-2-positive macrophages and macrophage count (n = 6). White arrows indicate MOMA-2-positive, DAPI-stained macrophages. ****P* < 0.0001 vs. sham group; ###*P* < 0.0001 vs. AAA-PBS group; n.s. *P* > 0.9999 vs. AAA-D123 group. **(E)** Representative microscopic images of in situ zymography. The section of lung served as a positive control and the other incubated with EDTA as a negative control. **(F)** Representative microscopic images of VVG staining and elastin break (n = 6). Blue arrows indicate disrupted elastic lamella. ****P* < 0.0001 vs. sham group; ###*P* < 0.0001 vs. AAA-PBS group; n.s. *P* > 0.9999 vs. AAA-D123 group. D123 indicates rCD93D123. D1 indicates rCD93D1. L indicates lumen. All scale bars represent 50 μm. Data are represented as mean values ± SEM and comparative statistical analyses were done by one-way ANOVA followed by multiple comparisons.

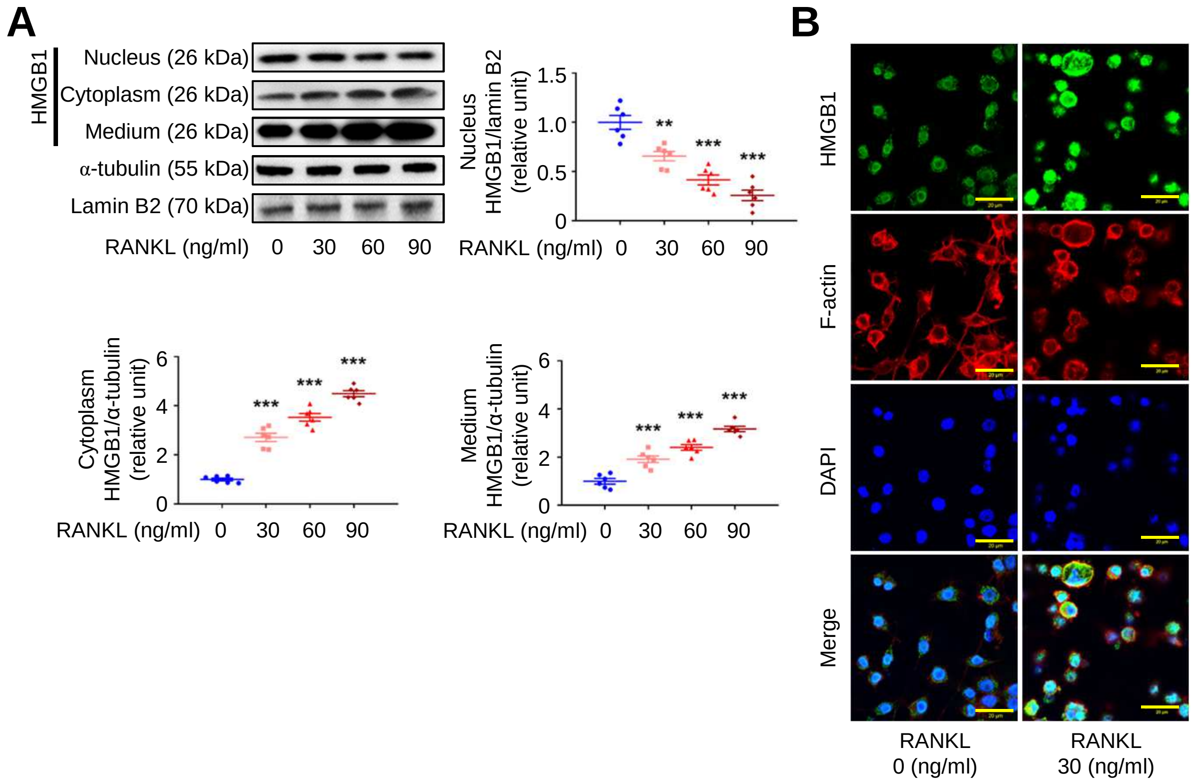

Figure S5. RANKL stimulates HMGB1 translocation and release in RAW264.7 macrophages. RAW264.7 macrophages were treated with indicated doses of RANKL for 1 day. **(A)** Representative western blot analysis and quantification of nucleus, cytoplasm, and medium HMGB1 (n = 6). For nucleus HMGB1/lamin B2 ***P* = 0.0020 vs. RANKL-negative group; ****P* < 0.0001 vs. RANKL-negative group. For cytoplasm HMGB1/α-tubulin and medium HMGB1/α-tubulin ****P* < 0.0001 vs. RANKL-negative group. **(B)** Representative immunofluorescence staining of HMGB1 and F-actin in RAW264.7 macrophages. All scale bars represent 20 μm. Data are represented as mean values ± SEM and comparative statistical analyses were done by one-way ANOVA followed by multiple comparisons.
